# Multi-site desmoplastic small round cell tumors are genetically related and immune-cold

**DOI:** 10.1038/s41698-022-00257-9

**Published:** 2022-04-04

**Authors:** Chia-Chin Wu, Hannah C. Beird, Salah-Eddine Lamhamedi-Cherradi, Melinda Soeung, Davis Ingram, Danh D. Truong, Robert W. Porter, Sandhya Krishnan, Latasha Little, Curtis Gumbs, Jianhua Zhang, Mark Titus, Giannicola Genovese, Joseph A. Ludwig, Alexander J. Lazar, Andrea Hayes-Jordan, P. Andrew Futreal

**Affiliations:** 1 Department of Genomic Medicine, The University of Texas MD Anderson Cancer Center, Houston, TX USA; 2 Department of Sarcoma Medical Oncology, The University of Texas MD Anderson Cancer Center, Houston, TX USA; 3 Department of Pathology, The University of Texas MD Anderson Cancer Center, Houston, TX USA; 4 Department of Genitourinary Medical Oncology, The University of Texas MD Anderson Cancer Center, Houston, TX USA; 5 Department of Surgical Oncology, The University of Texas MD Anderson Cancer Center, Houston, TX USA

**Keywords:** Sarcoma, Computational biology and bioinformatics

## Abstract

Desmoplastic small round cell tumor (DSRCT) is a highly aggressive soft tissue sarcoma that is characterized by the *EWSR1-WT1* fusion protein. Patients present with hundreds of tumor implants in their abdominal cavity at various sites. To determine the genetic relatedness among these sites, exome and RNA sequencing were performed on 22 DSRCT specimens from 14 patients, four of whom had specimens from various tissue sites. Multi-site tumors from individual DSRCT patients had a shared origin and were highly related. Other than the *EWSR1-WT1* fusion, very few secondary cancer gene mutations were shared among the sites. Among these, *ARID1A*, was recurrently mutated, which corroborates findings by others in DSRCT patients. Knocking out *ARID1A* in JN-DSRCT cells using CRISPR/CAS9 resulted in significantly lower cell proliferation and increased drug sensitivity. The transcriptome data were integrated using network analysis and drug target database information to identify potential therapeutic opportunities in EWSR1-WT1-associated pathways, such as PI3K and mTOR pathways. Treatment of JN-DSRCT cells with the PI3K inhibitor alpelisib and mTOR inhibitor temsirolimus reduced cell proliferation. In addition, the low mutation burden was associated with an immune-cold state in DSRCT. Together, these data reveal multiple genomic and immune features of DSRCT and suggest therapeutic opportunities in patients.

## Introduction

Desmoplastic small round blue cell tumors (DSRCT) were first described as a distinct entity in 1989^1^. It is highly aggressive and is considered a rare mesenchymal tumor composed histologically of small round blue cells of epithelial, muscular, mesenchymal, and neural differentiation admixed with prominent desmoplastic stroma^1,2^. DSRCT arises primarily in adolescents and young adults^3,4^ and has a striking male predominance (85–90%)^2,5^. Patients normally present with dozens or most often, hundreds of tumor implants in the abdominal cavity^2^. Owing to the extent and multi-site nature of the disease, chemotherapy, debulking, and radiation therapies has limited the effectiveness against DSRCT^6^.

DSRCT is characterized by the translocation, t(11;22)(p13;q12) which results in the *EWSR1-WT1* gene fusion^3^. WT1 is a tumor suppressor that contains four zinc finger DNA binding domains. The loss of the proximal zinc fingers of WT1 in the chimeric fusion protein converts WT1 from a transcriptional repressor to an activator and presumably initiates the oncogenic process in DSRCT^7,8^. Recent studies using cell and mouse models have demonstrated that the EWSR1-WT1 fusion can transform mouse embryonic fibroblasts and has pronounced effects on the proliferation, viability, and growth of DSRCT cells^9–11^. This transformation may be directed by several downstream targets of the EWSR1-WT1 fusion including growth factor/receptor genes (e.g. *IGF2*, *IGF1R*, *EGFR*, *FGFR4, PDGFA*, *VEGFR*), transcriptional regulators (e.g., *MYC*, *PAX2*), matrix cellular genes (e.g., *CDH1*, *CTGF*), and others (e.g., *AR*, *PARP*)^12–16^. Treatments directed at the EWSR1-WT1 fusion protein or downstream signaling pathways are not currently available. Some of the target genes may have therapeutic values in DSRCT, but require further evaluation.

Although patients all harbor the *EWSR1-WT1* driver mutation, there is substantial heterogeneity in treatment response and survival outcomes among DSRCT patients. Such heterogeneity may be due to secondary genomic alterations that are distributed differently across tissue sites. Owing to the rarity of the disease, limited genomic sequencing on single DSRCT specimens has been performed to identify critical genetic alterations other than the EWSR1-WT1 fusion. Analyses of sequencing panels, exomes, and genomes have identified recurrent secondary mutations in *AR, FGFR4*, *ARID1A*, *TERT*, *TP53*, *MSH3*, *HRAS*, *TOP2A*, *TOPO1*, *PTEN*, mucin genes, and others^17–19^. Genes related to mesenchymal cell differentiation or mesenchymal-epithelial reverse transition (MErT)/epithelial–mesenchymal transition (EMT) have been found to be altered as non-silent somatic mutations or gains in chromosome 1^20,21^. Copy number gains were seen in chromosomes 5 and 18, as well as copy number losses at 11p, 13q, and 22q^19,20^. Loss of the entire chromosome 6 where immune-regulatory genes are located have also been recorded^21^. However, the impact of these genetic alterations on DSCRT oncogenesis is unclear and little effort has been made to systematically characterize DSRCT lesions at various tissue sites. Such characterization could improve our understanding of the heterogeneity within the multi-site tumors, uncover genetic events underlying neoplastic initiation, and inform the development of targeted therapeutic strategies.

In the present study, we conducted exome and RNA sequencing on 22 DSRCT specimens from 14 patients, four of whom had multiple specimens. Extensive analyses of point mutations, copy number alterations, differential expression, and pathways were performed to identify secondary genetic alterations other than the EWSR1-WT1 fusion and pathways that may contribute to DSRCT progression. Phylogenetic analyses of multiple sites were used to determine whether the tumors arose independently or were related. To gain insights into the immunogenic potential of these tumors, we also examined the levels of immune infiltration using the RNA sequencing data. We also employed our network-based analytical approach to mine gene expression data to identify new EWSR1-WT1 fusion-associated gene targets potentially susceptible to therapeutic intervention.

## Results

We collected 22 frozen resections from 14 male patients and also included the patient-derived cell line JN-DSRCT for exome and transcriptome profiling (Table 1). Patient specimens were sampled from several areas where multiple tumor implants occur in 98% of DSRCT patients: the omentum, the peritoneum in the diaphragm region, and the peritoneum from the pelvic region around and between the bladder and rectum (Table 2). Four patients (AHJT2, 5, 13, 14) had two to five specimens taken from various tissue sites within the retroperitoneum including regions near the omentum and colon (Table 2). For each frozen tumor, exome sequencing was profiled with matching germline specimens (average tumor mean coverage = 228X; cell line = 320X) along with matched transcriptomes. As expected, the *EWSR1-WT1* fusion was detected in all of these specimens using RNA sequencing fusion callers.

**Table 1:** Clinicopathologic characteristics of 22 DSRCT samples from 14 patients.

Feature	Number of cases (%)
*Age of diagnosis*	
Range	7-52.9
Median	18.35
Gender/Race	
Female	0
Male	14 (100%)
White	10 (71.4%)
Hispanic	3 (21.4%)
Asian	1 (7.1%)
*Sample type*	
Intra-abdominal	12 (85.7%)
Extra-abdominal	2 (14.3%)
Pre-treatment (diagnostic)	8 (53%)
Post-treatment	6 (42.9%)
Pathology Type	
Primary	12 (85.7%)
Metastatic	2 (14.3%)
*Metastatic disease at Dx*	
Yes	10 (71.4%)
No	4 (28.6%)
Liver	4 (28.6%)
Liver, Lymph node	4 (28.6%)
Lymph node	1 (7.1%)
Lung	1 (7.1%)
*Treatment modalities*	
Chemo, surgery	5 (35.7%)
Chemo, surgery, radiation	8 (57%)
Not known	1 (7.1%)

**Table 2:** Information of samples from 14 patient and 1 cell line.

Patient ID	Sample ID	WT1_fusion	Tissue source
AHJT2	AHJT2a	Y	Not available
	AHJT2b	Y	Not available
AHJT3	AHJT3	Y	Not available
AHJT5	AHJT5a	Y	Not available
	AHJT5b	Y	Not available
AHJT6	AHJT6	Y	Not available
AHJT8	AHJ-T8	Y	Not available
AHJT9	AHJ-T9	Y	Not available
AHJT12	AHJ-T12a	Y	Colon
AHJT13	AHJ-T13a	Y	Omentum
	AHJ-T13b	Y	RUQ peritoneum
	AHJ-T13c	Y	Pelvic Peritoneum
AHJT14	AHJ-T14a	Y	Retro peritoneum
	AHJ-T14b	Y	Diaphragm
	AHJ-T14c	Y	Omentum
	AHJ-T14d	Y	Colon
	AHJ-T14e	Y	Pelvis
AHJT15	AHJ-T15	Y	Peritoneum
AHJT19	AHJ-T19	Y	Not available
AHJT20	AHJ-T20	Y	Peritoneum
AHJT21	AHJ-T21	Y	Omentum
AHJT23	AHJ-T23b	Y	Omentum
JN-DSRCT	JN-DSRCT	Y	DSRCT cell

### DSRCT has low mutation burden and few recurrently mutated cancer genes

**Fig. 1:**
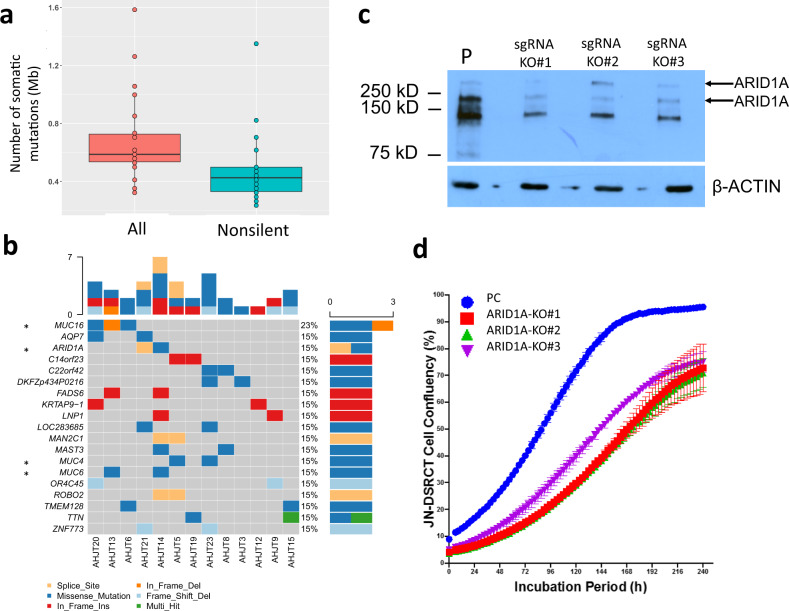
Mutation analysis. **a** Point mutation rate (number of mutations per Mb) in our DSRCT specimens. Boxplots show center line as the median, box limits as upper and lower quartiles of the mutation rates. **b** Landscape of recurrently mutated genes. Genes are shown according to patient, not specimen, so mutations in different specimens from the same patient are shown in the same column. Genes that are known to be associated with cancer are marked with “*”. The cancer gene annotations were compiled from the Sanger Cancer Gene Census in COSMIC. **c** Western blot demonstrating CRISPR/CAS9-mediated knockout of *ARID1A* using three different guide sgRNAs (KO#1, KO#2, KO#3) in JN-DSRCT. β-actin is used as a loading control. PC—parental line as a control. All blots derived from the same experiment and were processed in parallel. **d** Time course for the effect of *ARID1A* knockout results in significantly reduced proliferation (Confluence (%) is presented as the mean SD, *n* = 3 wells). Boxplots show center line as the median, box limits as upper and lower quartiles of the data. The source data of this figure is provided in the Source Data file.

Our DSRCT samples had a low mutation rate (median of 0.72 mutations per Mb), with a median of 23 non-silent mutations per tumor (Fig. 1a), which is similar to other fusion-driven sarcomas such as Ewing sarcoma^22,23^, synovial sarcoma^24^, and rhabdomyosarcoma^25^. Our observed mutation rate was similar to those observed in DSRCT by Devecchi et al. ^21^ and Slotkin et al. ^19^, but much lower than that seen by Bulbul et al. (4–8 mutations per Mb)^17^. Mutation signature analysis^26^ was not performed owing to the potential analysis biases resulting from the low mutation burden that was detected.

In total, 460 non-silent mutations from 378 genes were identified in our patient cohort (Supplementary Data 1a). Of these genes, 19 were recurrently mutated in at least two patients, but most were not known cancer genes (Fig. 1b). Several of these recurrently mutated genes were identified in previous studies and may contribute to the development of DSRCT. First, three recurrently mutated mucin genes (*MUC16*, *MUC6*, *MUC4*) were identified in our cohort. In diverse types of human cancers, mucins deregulate multiple pathways, such as cell adhesion and Wnt pathways, to promote cancer cell growth and migration^27^. Mutations in mucin genes (e.g. MUC19) were also identified in two of the six patients in the cohort studied by Devecchi et al.^21^. In addition, two patients each harbored one mutation in *ARID1A*: one missense change in the BAF250_C domain and one splicing site mutation near the AT-rich interaction domain 1 A. Inactivating mutations in *ARID1A* are recurrent in other studies: 11% of 85 DSRCT patients, 7% of 68 DSRCT patients, with other case reports where one deleterious mutation and one variant of uncertain significance have also been observed^18,19,21,28^. ARID1A is a subunit of the SWI/SNF epigenetic regulator that is frequently altered in cancer. Given the prevalence of *ARID1A* mutations in DSRCT, we used CRISPR/CAS9 to knock out *ARID1A* in the JN-DSRCT cell line. All three guides that were used resulted in near ablation of ARID1A protein (Fig. 1c). Although there was no apparent alteration of cell morphology with the knockout line, cell proliferation was significantly reduced (Fig. 1d).

Given the low prevalence of gene-level mutations, we interrogated whether pathways were recurrently altered by mapping all the mutated genes to pathways. There was significant enrichment in cancer-associated pathways including PI3K/AKT signaling, cell adhesion, cell proliferation, and angiogenesis pathways (Supplementary Data 2). Based on findings by others, these mutations would be expected to facilitate EWSR1-WT1-mediated oncogenesis. For example, the PI3K/AKT/mTOR pathway is constitutively activated in DSRCT and mTOR and kinase inhibitors can achieve a stable response in DSRCT patients^29^. We also identified several mutated genes associated with cell adhesion in all patients. These mutations may work together with EWSR1-WT1 in transforming and promoting cell mobility and cancer progression.

### Most copy number changes occur at the chromosome arm or whole chromosome level

**Fig. 2:**
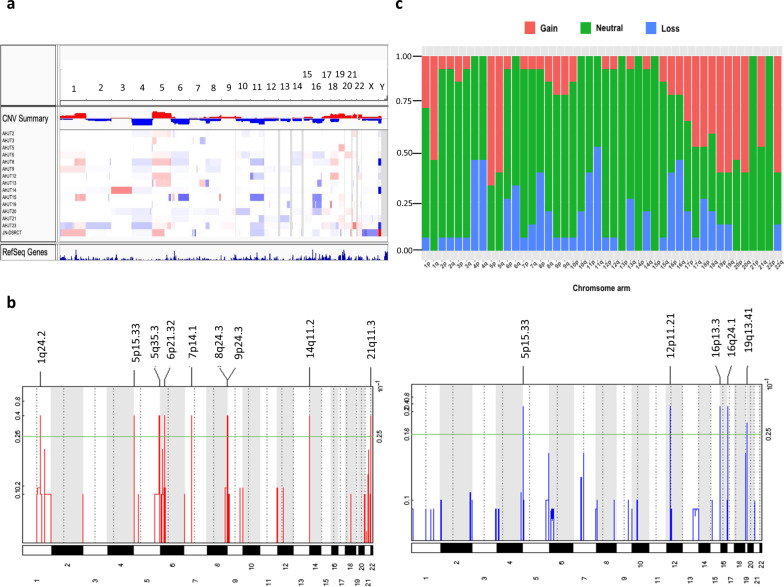
Identified SCNAs in the DSRCT patients. **a** the SCNA profiles of all the 14 patients (copy number profiles of samples from the same patients were merged by aligning chromosome segments across samples of the same patient and then assigning the mean of copy number changes to the overlapping chromosomal segments and keeping the copy number changes of those sample-specific segments). **b** Focal copy number alterations identified by GISTIC across the 14 patients. GISTIC *q*-values (*y*-axis) for copy amplification (left, red) and deletion (right, blue) peaks were plotted across the chromosomes. The significance levels of focal events are shown as green lines. **c** Frequency of significant arm-level SCNAs identified by GISTIC. Arm-level SCNAs were considered significant with GISTIC *q* < 0.05.

To delineate recurrent somatic copy number alterations (SCNAs) detected from the exome data across the 14 patients, we first combined SCNA segments of samples from the same patients (see Methods). The majority of these SCNAs involved entire chromosome arms or whole chromosomes (Fig. 2a), with very few significant focal events (Fig. 2b). Several arm-level and whole chromosome SCNAs were recurrent in more than half of the patients (Fig. 2a, c). Similar to the findings of Devecchi et al. ^21^, gain of chr1q and loss of chr6q were present in 55% and 30% of our patients, respectively. We also identified other recurrent gains in chromosomes 5, 17, 18, 19, 20, and 22 and recurrent losses in chromosomes 4, 11, and 16. Beroukhim et al. ^30^ compared arm-level SCNAs from 26 cancer types, including sarcomas: synovial sarcoma, gastrointestinal stromal tumor, leiomyosarcoma, pleomorphic liposarcoma, malignant fibrous histiocytoma (undifferentiated pleomorphic sarcoma), and dedifferentiated liposarcoma and found that cancer types from related developmental lineages tend to share arm-level SCNAs. Similar to these sarcomas, our DSRCT samples were enriched in gains in chromosomes 1q, 5, 19, 20, and 21q, and loss of chr11 were also enriched in our DSRCT cohort.

The large number of genes encoded by entire chromosome arms and whole chromosomes make it challenging to determine which SCNA genes contributed to tumor formation^30^. Thus, we integrated significantly differentially expressed genes between DSRCT samples and adjacent omentum samples with recurrent SCNA genes to prioritize those with higher potential impact (see the Methods). We identified 182 upregulated genes and 300 downregulated genes with recurrent copy number gains or losses in at least four patients (Supplementary Data 3). These 482 genes were associated with pathways related to cellular apoptosis, proliferation, cell motility, and metastasis such as the Wnt pathway and stathmin pathways (Supplementary Data 4). By overlapping the 482 genes with genes whose promoter regions were peak binding sites of WT1 by Chip-seq^16^, we also identified 6 of the 482 genes that may be direct targets of the EWSR1-WT1 fusion: *CELF3* (chr1q21.3); *UBQLN4* (chr1q22), *RYR2* (chr1q43); *UBTD2* (chr5q35.1); *POGZ* (chrq21.3); and *EZF4* chr16q22). Two of these genes, *CELF3* and *RYR2* have roles in muscle and may partially explain that DSRCT is muscle-like.

### Multi-site tumor samples from the same patients are closely related

**Fig. 3:**
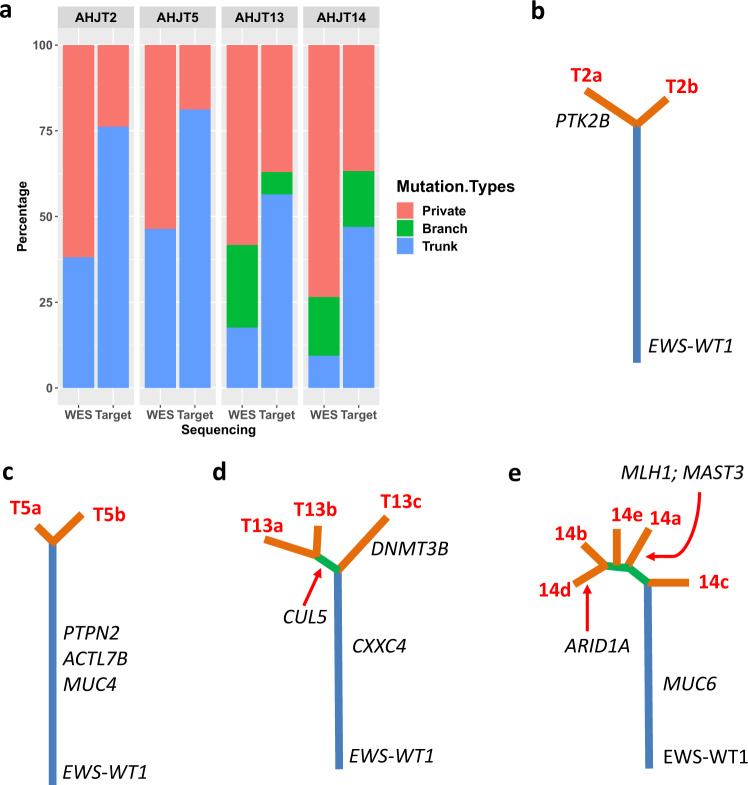
Tumor Heterogeneity analysis of DSRCT samples. **a** Distribution of trunk, branch, and private branch mutations defined by WES and targeted deep sequencing. **b**–**e** Mutation phylogenetic trees of patients AHJT2T, -5, -13, and -14. Blue, green, and orange lines of the phylogenetic trees represent trunk, branch, and private branches respectively. The mean allelic frequency to trunks of the four phylogenetic trees are respectively 0.209, 0.234, 0.169, and 0.156 while the mean allelic frequency to branches (including privates) of the trees are respectively 0.126, 0.125, 0.142, and 0.121.

Most DSRCT patients present with advanced-stage, multi-site tumor burden that can be highly heterogeneous. Given that intra-tumor heterogeneity can alter cancer progression, recurrence, and therapeutic resistance, we sought to better define the relatedness among multiple tumor sites in individual DSRCT patients^31,32^. To achieve this, phylogenetic trees were constructed by comparing somatic mutations from different tissue sites for 4 DSRCT patients (AHJT2, 5, 13, and 14) for whom we had samples from multiple sites. Clonal inference analysis was not applied to phylogenetic inference due to the low mutation burden. For each patient, the trunk of the phylogenetic tree represents mutations shared by all tissue sites; the large branches show mutations seen in more than one site; and the smaller branches display mutations that are unique to each site. On average, 28% of the exonic mutations were shared by all tissue sites, forming the trunks of the phylogenetic trees (Fig. 3a). This percentage was then increased to an average of 60% using deep targeted sequencing of all WES mutations that were detected in at least one sample of a given patient. Thus, deeper sequencing revealed that many of the branch and private branch mutations detected by WES were indeed present and detectable in all samples of a given tumor (i.e., trunk mutations), revealing that the tumors were closely related to one another (Fig. 3a). Again, this demonstrates how considerable sequencing depth is necessary to characterize the phylogenetic tree accurately^32^. Mutations validated by deeper sequencing were used to build phylogenetic trees of the four patients (Fig. 3b–e). We then examined whether any of the non-silent mutated genes in the trunks were known cancer-linked genes. No such genes were detected in patient 2. However, missense mutations in known cancer genes were found: *PTPN23* (p.R1599W) and *ACTL7B* (p.T12M) for patient 5, *CXXC4* (p.S150W) for patient 13, and *MUC6* (p.H1446Y) from patient 14. None of them were recurrently mutated in the entire cohort. In addition, *ARID1A* and *MAST3* that were located within the branches of patient 14 are among the recurrently mutated cancer genes. Therefore, we found no point mutation consistently required for early DSRCT oncogenesis other than the EWSR1-WT1 fusion.

We also further explored the tumor heterogeneity of DSRCT using SCNA data derived from WES. Notably, the SCNA profiles of multiple samples from individual patients were very similar (Supplementary Fig. 2–6), and different samples from individual patients were significantly correlated (Supplementary Fig. 7). Most arm-level or whole chromosomes SCNAs were shared across tissue sites from individual patients (i.e., trunk SCNAs) (Supplementary Fig. 2–6). For example, all samples from patient 14 had gains in chr3 and chr7q and loss in chr16q. Gains in chr5 were present in all the samples from patients 2 and 13. All the samples of patient 5 shared the gain in chr19. Few arm-level SCNAs were observed in an individual sample from a patient. For example, losses of chr10q and 17p were only observed in sample T2a of patient 2. Loss in chr18 was observed in the only sample T13a from patient 13. Similar to the phylogenetic trees built using mutation data, those based on SCNA profiles (Supplementary Fig. 8) also had long trunks with short branches. Therefore, our phylogenetic tree analyses using both point mutation and SCNA data suggest that different tumors from the same DSRCT patients have a shared origin and are closely related.

### DSRCT is an immune-cold tumor type

**Fig. 4:**
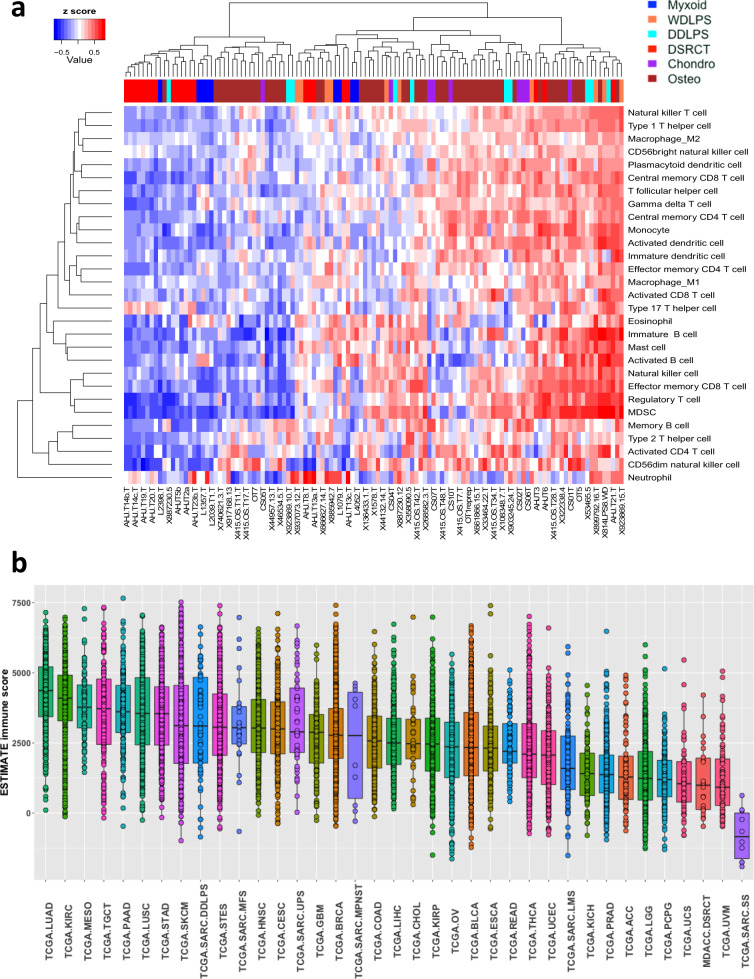
Immune filtration analysis using RNA-seq data. **a** Immune cell profile across samples of DSRCT and other types of sarcoma samples from MD Anderson (MDACC). Immune cell profiles were characterized using single-sample GSEA scores of immune cell gene sets. DDLPS, dedifferentiated liposarcoma; Osteo, osteosarcoma; WDLPS, well-differentiated liposarcoma; Myxoid, myxoid liposarcoma; Chonro, chondrosarcoma. **b** ESTIMATE immune filtration scores in our DSRCT samples and samples of different TCGA tumor types. Each boxplot shows center line as the median, box limits as upper and lower quartiles of the immune filtration scores for each TCGA tumor type. Abbreviations of the tumor types are defined in Supplementary Table 2.

To gain insights into the immunogenic potential of DSRCT, we first applied single-sample gene set enrichment analysis (ssGSEA) for various immune cell types^33^ to DSRCT and other types of sarcoma samples that have been sequenced at our institution. We also compared the composition of the infiltrating immune cells across samples. The majority of our DSRCT samples belonged to the immunologically “cold” group that lacks most types of immune cells (Fig. 4a). This is consistent with synovial sarcomas, another fusion-driven sarcoma that also has few infiltrating immune cells than other types of sarcomas^34^. Neutrophils and T helper 17 cells were abundant in a subset of patients, but the significance of this finding is unclear.

To further our understanding of the DSRCT immune microenvironment in a global context, we took the immune infiltration scores of our DSRCT samples (MDACC.DSRCT) as generated by ESTIMATE^35^ and compared them with other tumor types profiled in The Cancer Genome Atlas (TCGA). The immune infiltration scores of DSRCT were low and similar to those of tumor types for which immune checkpoint blockades inhibitors (ICI) have low efficacy, such as low-grade glioma (TCGA.LGG), prostate cancer (TCGA.PRAD), and uveal melanoma (TCGA.UVM) (Fig. 4b). Within this pan-cancer context, we found that the median ESTIMATE scores of our DSRCT samples were the lowest among cancer types. None of our DSRCT samples had ESTIMATE immune scores within the highest quartile among all the samples. Compared to other sarcoma types, the median ESTIMATE score of our DSRCT samples was similar to that of another fusion-driven sarcoma, synovial sarcoma (TCGA.SARC.SS). Still, it was significantly lower than those of two soft tissue sarcoma subtypes, dedifferentiated liposarcoma (TCGA.DDLPS) and undifferentiated pleomorphic sarcoma (TCGA.UPS), where ICI are active in a proportion of patients^36^. These results suggest that most of our DSRCT specimens did not have sufficient immune cell infiltrate to elicit meaningful responses to ICI.

### DSRCT is distinct from other sarcomas

**Fig. 5:**
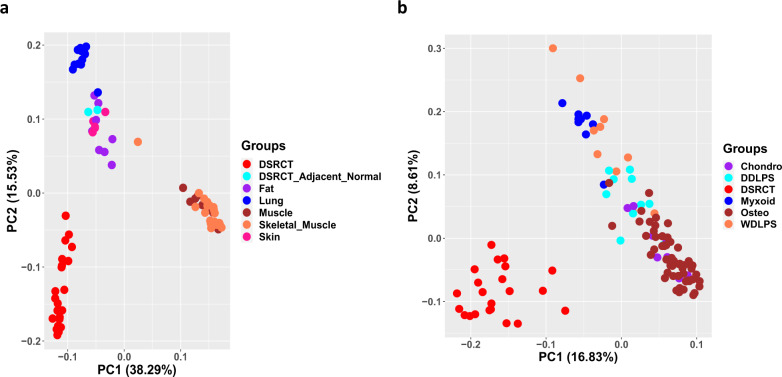
Principal component analysis (PCA) analysis. **a** Gene expression comparison between DSRCT samples and normal samples of several tissues in MDACC. **b** Gene expression comparison between DSRCT samples and other types of sarcomas.

We sought to determine whether there were any similarities in the gene expression patterns of DSRCT and those of various types of normal tissues as well as other types of sarcoma tissue. Principal component analyses showed that DSRCT differs substantially from adjacent normal tissues and other sarcoma subtypes (Fig. 5a, b). DSRCT samples were clustered independently, closer to liposarcoma than to osteosarcoma and chondrosarcoma (Fig. 5b). Because the origin of DSRCT is unknown, this result suggests that DSRCT may be more closely related to adipocyte tissues than to myocyte or osteocyte tissues. To identify genes that may be deregulated in DSRCT, we compared gene expression between DSRCT samples and the two adjacent omentum samples. In total, 7,480 differentially expressed genes (*P* < 0.05 and fold changes >1.5) were found (Supplementary Fig. 9), some of which may not be deregulated because of the *EWSR1-WT1* fusion. Therefore, we integrated this differential gene expression analysis with the network analysis to infer genes and pathways that are specifically associated with the *EWSR1-WT1* fusion in DSRCT, described below.

### EWSR1-WT1 fusion-associated pathways and therapeutic targets

**Fig. 6:**
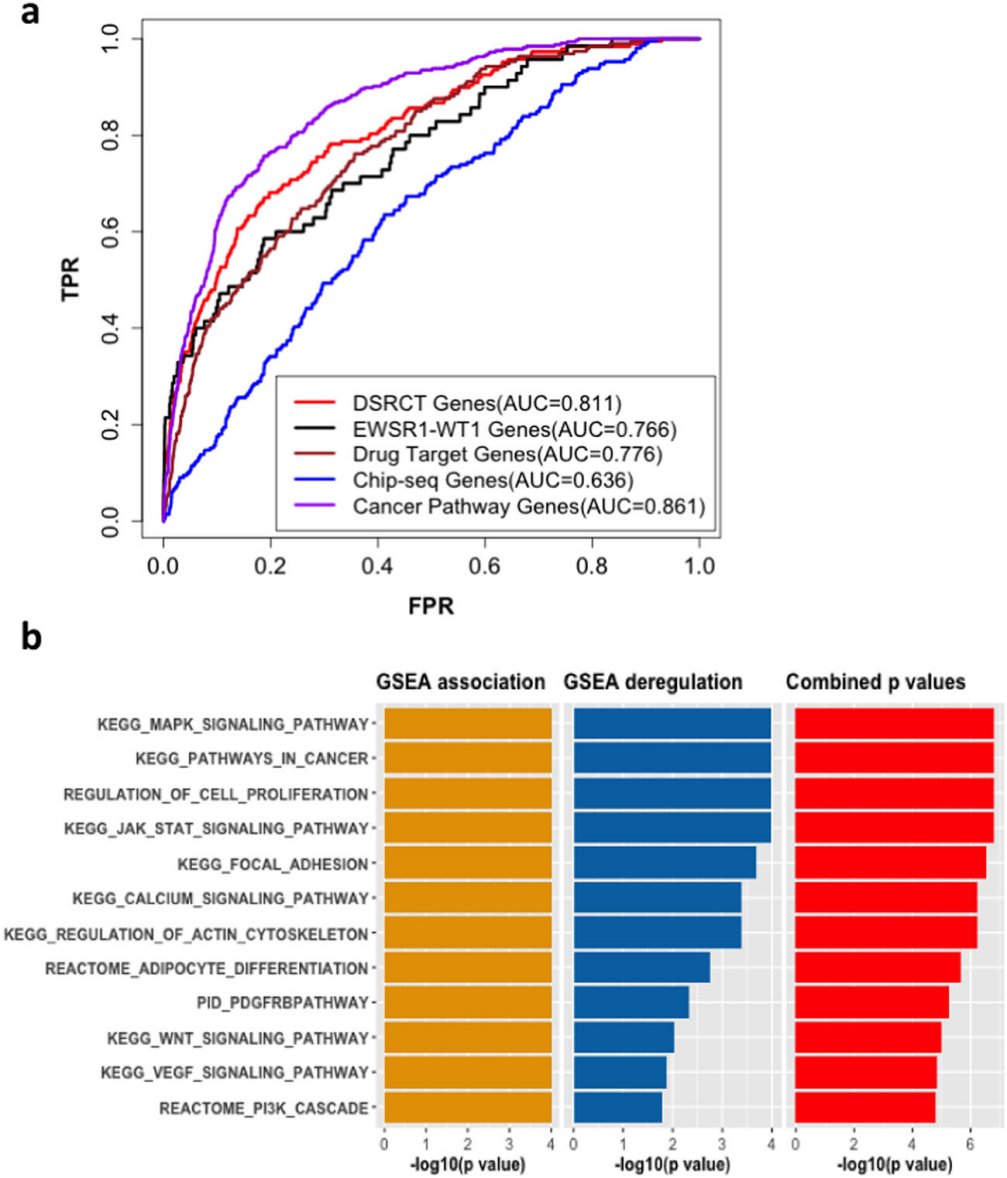
Prediction of genes and pathways associated with EWSR1-WT1. **a** Prediction evaluation using four benchmark gene sets: 74 genes cited with EWSR1-WT1 in the literature (denoted EWSR1-WT1 genes), 201 genes cited with DSRCT in the literature (denoted DSRCT genes), 194 target genes of compounds that have been tested in clinical trials or used for the treatment of DSRCT (denoted drug targets), 268 genes whose promoter regions were peak binding sites of WT1 by ChIP-Seq (indicated as ChIP-Seq genes), and 328 genes categorized in the KEGG cancer pathways (denoted cancer pathway genes). Genes in these four gene sets were treated as positive instances, and the remaining genes were treated as negative instances. TPR indicates true positive rate; FPR, false positive rate; and AUC, area under the ROC curve. **b** Bar plot of some statistically significant pathways that are associated with EWSR1-WT1 in our prediction. Each bar represents the statistical significance of a given pathway in GSEA association analysis (pathway analysis of the network association), GSEA deregulation analysis (pathway analysis of gene expression deregulation), or combination analysis. The statistical significances are presented in the graph as -log10 (*p*-value).

Although most fusion transcription factors are considered ‘undruggable’, recent evidence in Ewing sarcoma indicates that direct antagonists of EWSR1-WT1 may eventually be discovered. In the meantime, a more tractable approach is to identify interacting proteins that rely on the EWSR1-WT1 fusion to sustain tumor growth and survival. Therefore, we used integrative network analysis to identify genes and pathways that are associated with the EWSR1-WT1 fusion in DSRCT (see the analysis flowchart in Supplementary Fig. 10A). First, we applied our computational network approach, FusionPathway^37^, to identify genes and pathways that are related to the EWSR1-WT1 fusion in a gene network. The first step in this approach is to use each protein domain of the fusion to predict it’s protein-protein and transcription regulatory interactions. These molecular interactions are then used to infer associated genes and pathways of the fusion. This network analysis inferred the functional association of each gene with the fusion (the network association result is listed in Supplementary Data 5). Several known downstream targets of EWSR1-WT1, such as *AR*, *MYC*, *EGFR*, *CDH1*, *CTGF*, *MTOR*, *IGF1R*, and *PDGFRA*^11–13^ were among the top 10% genes in our prediction list (Supplementary Data 5). To comprehensively evaluate our predictions, we used several benchmark gene sets (see the Methods), including EWSR1-WT1 fusion-related genes, DSRCT-related genes, and cancer pathway genes as well as a set of genes whose promoter regions were found to be peak binding sites of WT1 by ChIP-Seq^38^. The receiver operating characteristic analysis (Fig. 6a) and one-side Mann–Whitney–Wilcoxon tests (Supplementary Fig. 11) showed that most of these benchmark genes were among the top hits on our prediction list. We then used gene set enrichment analysis (GSEA)^36^, which can evaluate the genes of a pathway for their distribution in the ordered gene list of our prediction, to identify pathways associated with the EWSR1-WT1 fusion in the gene network (termed GSEA association analysis).

Since the gene network used in the prediction contains molecular interactions across different cell types, not all predicted associated pathways (GSEA association analysis) are deregulated by EWSR1-WT1 in DSRCT^37^. Therefore, we also applied GSEA to determine the deregulation level of each associated pathway in the network prediction using the differential gene expression data between DSRCT and the adjacent omentum samples (termed GSEA deregulation analysis). We then used the truncated product method [59] to combine the *p*-values of the GSEA association analysis mentioned above with the GSEA deregulation analysis for each pathway to identify pathways that were not only highly associated with the EWSR1-WT1 fusion in the network analysis but also significantly deregulated in DSRCT (Fig. 6b, Supplementary Data 6). Several known pathways of DSRCT were identified, such as the PI3K, JAK-STAT, WNT, MYC, and MAPK signaling pathways (Fig. 6b)^13,14,39^. In addition, several top-ranking pathways in our prediction list were those whose expression was altered following knockdown of EWSR1-WT1^10^, such as PI3K-AKT, focal and cell adhesion, calcium signaling, and TGFB pathways (Supplementary Data 7). This result further supports our pathway prediction. Of note, two of the most significantly enriched pathways were the cell adhesion and calcium signaling pathways. Cell adhesion and migration-related pathways were also considerably deregulated in DSRCT as compared with other types of sarcoma (Supplementary Fig. 12), suggesting that DSRCT cells may have a greater capacity to migrate throughout the abdominal cavity to form the hundreds of tumor implants that are found at diagnosis^2^. Calcium signaling may also provide insights into tumor initiation, angiogenesis, progression, and metastasis^40^.

Our previous study demonstrated that our network approach identifies agents that target fusion-associated pathways in two sarcomas, Ewing (EWSR1-FLI1) and myxoid liposarcoma (FUS-DDIT3). These predictions were validated by our drug screens on Ewing and myxoid liposarcoma cells^37^. In like manner, we collected 194 target genes of 52 drugs that have been in clinical trials for treating DSRCT patients to validate our prediction in DSRCT (see Methods; Supplementary Data 8). The majority of these 194 genes were highly associated with the EWSR1-WT1 fusion in our prediction (Fig. 6a; AUC = 0.775). We further mapped the top 15% of genes in our network prediction to drugs that they interacted with based on the DGIdb database^41^. We retained those genes that were significantly upregulated in DSRCT samples as compared with adjacent normal omentum samples (see the analysis flowchart in Supplementary Fig. 10B). In total, we identified 28 genes that could be targeted by inhibitors or antagonists (Supplementary Data 9). These genes included several known target genes in DSRCT, such as *AR*, *KDR*, and *PDGFA*. Some inhibitors of these targets have anti-cancer effects in other cancer types and could provide potential therapeutic benefits for DSRCT patients (Table 3). For instance, *FGFR4* is amplified in 8% of 83 DSRCT patients in one study and amplified in 7% of 67 DSRCT samples in another study, resulting in high levels of expression as compared to other fusion-driven sarcomas^18,19^. *FGFR4*, which is involved in regulating cell proliferation, cell differentiation, and cell migration, is induced by the EWSR1-WT1 fusion in DSRCT cell lines, possibly through binding of EWSR1-WT1 within its coding regions^16,42^. Its inhibitors, such as lenvatinib, have been used to treat thyroid, hepatocellular, renal, and adenoid cystic carcinomas^43^. *ERBB2* (erb-b2 receptor tyrosine kinase 2, HER2/neu) is involved in some signaling pathways, such as MAPK and PI3K pathways. Overexpression of *ERBB2* was also seen in 7 of 18 DSRCT cases^44^, and a recent study also showed that treatment of DSRCT cell lines with ERBB ligands could activate ERBB2 and stimulate DSRCT cell growth^45^. Therefore, the *ERBB2* inhibitor, pertuzumab, could be used to treat DSRCT patients. *PARP1* encodes a chromatin-associated enzyme involved in the regulation of differentiation, proliferation, tumor transformation, and DNA damage. In one recent study, *PARP1* expression was observed in 100% of DSRCT tumor samples (*N* = 16) and its inhibitor, olaparib, reduced DSRCT cell viability and migration^38^.

**Table 3:** Selected potential therapeutic targets associated with *EWS-WT1* in our prediction.

Compound	Selected target genes	Log2 Fold changes in expression (DSRCT vs Adjacent Normal)	Percentile rank (%) in our prediction	Application
Enzalutamide	*AR*	1.91	0.0096	Prostate cancer
Apelisib	*PI3K eg. PIK3C3*	0.62	0.00013	Multiple solid tumors
Enzastaurin	*GSK3B*	0.97	0.21	Lung cancer^39^
Pertuzumab	*ERBB2*	1.96	1.25	DSRCT PDX^45^
Vorinostat	*HDAC6*	1.22	1.76	Sarcoma cell lines^46^
Olaparib	*PARP1*	1.56	1.9	DSRCT in vitro and in vivo^38^
Regorafenib	*RAF1*	0.97	2.52	Phase II trial in sarcoma^47^
Lenvatinib	*FGFR4*	5.01	2.75	Solid tumors^43^
Palbociclib	*CDK6*	1.40	10	Sarcoma^48^

**Fig. 7:**
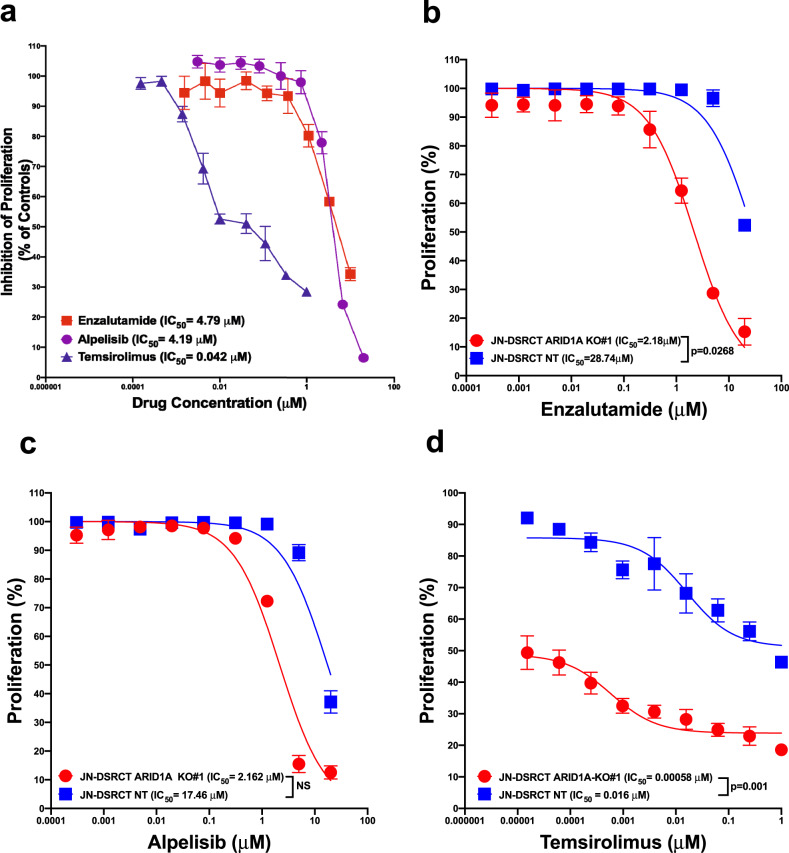
Inhibition of JN-DSRCT proliferation. **a** Serial dilution of the PI3Kα inhibitor apelisib (20 mM), mTOR inhibitor temsirolimus (1 mM), and AR inhibitor enzalutamide (20 mM) were given in triplicate and imaged every four hours for 5 days (enzalutamide and alpelisib) and 9 days (temsirolimus). This graph was based on cell mean a percentage of untreated cells (control DMSO) corresponding to the percentage inhibition of proliferation on the last day of imaging. **b**–**d** Knockout of ARID1A in JN-DSRCT using the sgRNAs (KO#1) enhances sensitivity to enzalutamide (**b**), alpelisib (**c**), and temsirolimus (**d**) as measured by a cell-based proliferation assay in a time-dependent manner using Incucyte. NT = parental control. The error bars in the plots represents standard deviation. The IC50 values corresponding to the NT cells in all the assays vary widely because of their different endpoint times: (**b**) (240 h), (**b**, **c**) (192 h), and (**d**) (144 h). It might also be due to the noise, and mainly, these proliferation assays had been run for an extended time (6–10 days).

Taken together, the enriched pathways based on both the somatic point mutations (Supplementary Data 2) and the integration of network prediction with differentially expressed genes (Fig. 6b; Table 3) implicated the PI3K/mTOR pathways would be an important pathway in DSRCT. Therefore, we tested the effect of the PI3Kα inhibitor alpelisib and the mTOR inhibitor temsirolimus on the proliferation of the JN-DSRCT cells (Fig. 7a). Given the overexpression of AR in DSRCT, we also examined the therapeutic potential of the androgen receptor inhibitor enzalutamide (Fig. 7a). For all three compounds, inhibition of proliferation was seen with IC50 values under 5 µM. Temsirolimus had the most pronounced effect with an IC50 value of 0.042. Since we observed reduced proliferation when ARID1A was knocked out in JN-DSRCT, we then treated the ARID1A knockout cells with each of these drugs (Fig. 7b–d). Temsirolimus had a stronger cytostatic effect on ARID1A knockout cells than it did on the parental line.

## Discussion

We conducted comprehensive genomic and transcriptomic profiling of DSRCT patient specimens to understand the molecular bases of DSRCT and to explore therapeutic opportunities for patients. The point mutation rate in our exome data was low, with very few recurrently mutated cancer genes detected, similar to that which was seen in DSRCT by Devecchi et al. ^21^, Slotkin et al., and in other fusion-driven sarcomas such as Ewing sarcoma^22,23^, synovial sarcoma^24^, and rhabdomyosarcoma^25^. We detected several mutated cancer genes involved in PI3K-Akt signaling, cell adhesion, and cell proliferation pathways that were also identified in other DSRCT studies^10,39^, such as mucin genes and *ARID1A*. Our multiple-site mutation analysis indicated that most of these secondary mutations in cancer genes were subclonal (i.e., branch mutations). Several were trunk mutations, but none were recurrently mutated in the entire cohort. These results support the view that the *EWSR1-WT1* fusion is the likely perpetrator of tumor development, which is often the case for other fusion-driven cancers.

DSRCT has recurrent secondary mutations in *ARID1A*, including a truncating mutation (p.Q1639*) in one case^21^, a variant of unknown significance in another case^28^, inactivating mutations in 11% of 83 patients, and inactivating and deep deletion in 6% of 68 patients in another study^18,19^. ARID1A is a subunit of the SWI/SNF epigenetic regulator and orthologous BAF complex that can be a tumor suppressor or oncogene depending on context and timing, even in the same tumor type^46^. This may be the reason why knocking out ARID1A reduces proliferation as it may be an oncogene early on and then a tumor suppressor later. This may be supported by our observation that *ARID1A* was a subclonal mutation in our multiple-site mutation analysis and thus occurring later during tumor evolution.

Most of the SCNAs in our DSRCT samples spanned entire chromosome arms or entire chromosomes. Mitotic failure is believed to cause chromosome mis-segregation and induce large deletions and duplications^47^. EWS may influence the mitosis cellular process^48,49^ while WT1 may also interact with MAD2 and regulates mitotic checkpoint function^50^. Thus, the arm- or whole chromosome level copy number alterations in DSRCT may be due to the effects of the *EWSR1-WT1* fusion. Our network association analysis identified pathways related to mitosis and chromosome segregation as significantly associated with the EWSR1-WT1 fusion (Supplementary Data 6). However, these pathways were not significantly deregulated in DSRCT as compared with adjacent normal samples. Therefore, this association warrants further validation in other DSRCT cohorts, cell lines, and mouse models. In addition, several arm- and whole- chromosome level SCNAs alter the expression of pathways associated with cell proliferation, cell motility, and metastasis.

DSRCT is often diagnosed at a late stage, after tumors have already become large and spread to other parts of the body, mainly in the abdomen. To clarify whether multiple masses arose independently or have a shared origin, we constructed mutation and SCNA phylogenetic analysis for four patients for whom multi-site sampling were available. We observed that all samples had identical EWSR1-WT1 fusion breakpoints and that most mutations and SCNAs occurred within the trunks of the phylogenetic trees. No recurrently mutated genes other than the EWSR1-WT1 fusion occurred within the trunks. This suggests that all of the masses from individual patients were closely related, with homogeneous mutation profiles and likely developed from a single lesion where the EWSR1-WT1 fusion was the main driver of tumor initiation. This is consistent with the general belief that fusion-positive sarcomas are driven mainly by the fusion oncoprotein with few other genomic alterations, whereas fusion-negative sarcomas have higher genetic heterogeneity^51^. Although no significant genetic heterogeneity was observed in samples from individual patients, other types of heterogeneity may exist. For instance, epigenetic heterogeneity was observed in Ewing sarcoma^52^, which may be influenced by cancer stem cell state and/or their interaction with the tumor microenvironment^53^. Ewing sarcoma also has transcriptomic heterogeneity that was seen using single-cell RNA sequencing^54^. Therefore, additional analyses of methylation, chromatin immunoprecipitation, and single-cell transcriptomes will enhance our understanding of DSRCT heterogeneity and its clinical impact on patients.

Our network approach^37^ predicted genes and pathways associated with the *EWSR1-WT1* fusion. We satisfactorily validated our predictions using several benchmark gene sets, including *EWSR1-WT1* fusion-related genes, DSRCT-related genes, and genes whose promoter regions were identified as peak binding sites of WT1 by ChIP-Seq analysis^16^. Cell adhesion and calcium signaling were among the top predicted pathways and were differentially expressed in DSRCT as compared to other types of sarcoma and adjacent normal tissues. These pathways were also enriched in the list of perturbed genes after knockdown of the EWSR1-WT1 fusion in JN-DSRCT cells (Supplementary Data 7)^11^. Perhaps, *EWSR1-WT1* enables DSRCT tumor cells to utilize these pathways to disperse within retroperitoneal tissue from the peritoneal fluid, resulting in the hundreds of tumor implants in the abdominal cavity that are presented at diagnosis^2^.

The *EWSR1-WT1* fusion has been a problematic therapeutic target. Therefore, we integrated drug target database information with our top predicted genes in the network analysis to identify new targets and their associated compounds. Our prediction was highly concordant with 194 target genes of 52 compounds that have been tested in clinical trials for DSRCT treatment. Several potential therapeutic targets in DSRCT were identified, including AR and PI3K. We evaluated the activity of the AR inhibitor enzalutamide, PI3K inhibitor alpelisib, and mTOR inhibitor temsirolimus on JN-DSRCT cells. There was potential potency of all of these drugs, with the highest sensitivity of cells to temsirolimus. The results support the prediction of our integrative network analysis that indicates the PI3K/mTOR pathway would be therapeutic targets in DSRCT (Supplementary Data 6)^11^. Therefore, it is advantageous to combine bioinformatic predictions with patient profiling and in vitro data to identify viable therapeutic options for DSRCT patients.

Similar to other types of fusion-driven sarcomas such as synovial sarcoma, our DSRCT samples had lower overall immune infiltrate than other tumors types, especially those against which ICI are effective. This low level of immune infiltrate may be associated primarily with the low mutation burden in DSRCT. Therefore, as with other types of immunologically cold tumors, the immune response may require stimulation with vaccine, CAR T-cell, or NK cell-based therapies to elicit an immune response. For instance, D’Angelo et al. ^55^ demonstrated that adoptive T-cell therapy targeting the NY-ESO cancer-testis antigen elicits antitumor responses in 50% of treated synovial sarcoma patients. We also found that neutrophils and T helper 17 cells in our DSRCT samples were at levels comparable to other sarcomas. Neutrophils may suppress the proliferation of T-cells by depriving them of arginine through their high levels of the arginine transporter, arginase^56^. Arginase levels were higher in our DSRCT samples than in other sarcoma subtypes (*P* < 1 × 10^−4^, FDR < 0.001). However, whether neutrophils express these to render them immunosuppressive remains to be seen.

In summary, our findings demonstrate that multi-site tumors from individual DSRCT patients have a shared origin and are closely related. The EWSR1-WT1 fusion is almost certainly a major driver in the early development and dispersion of DSRCT tumors. Therefore, until compounds that directly inhibit the EWSR1-WT1 fusion are developed, the best short-term strategy to eradicating this aggressive pediatric sarcoma is indirect targeting of pathways that the EWSR1-WT1 fusion relies on to mediate tumor growth and survival. Although a DSRCT-specific neoantigen remains to be identified, the immune-cold state of the tumor offers an initial glimpse into some of the challenges investigators might face as they evaluate immunotherapeutic strategies.

## Methods

### Samples

Patients provided their written informed consent for their samples to be used and the study was approved by MD Anderson’s Institutional Review Board. We collected 22 frozen DSRCT specimens from 14 patients for analysis. The sample information and patient characteristics are detailed in Supplementary Table 1

### Whole exome sequencing and mutation analysis

DNA of the frozen samples were extracted using the frozen tissue protocol from the QIAamp DNA Mini kit (Qiagen, Inc., Valencia, CA). DNA samples were submitted for 76 bp short-read paired-end whole-exome sequencing on Illumina HiSeq 2000 (Illumina, Inc., San Diego, CA) after SureSelect Human All Exon V4 library preparation (Agilent Technologies, Inc., Santa Clara, CA). For each sample, the DNA reads were mapped to the hg19 reference genome using BWA-MEM. MuTect was then used to call somatic mutations from DNA reads of tumor samples against their matched normal sample, and Pindel was used to call indels. High-quality variants were defined as those with a minimum tumor read depth of ≥30, minimum matched normal read depth of ≥15, and minimum alternate allele frequencies in the tumor and normal as ≥0.05 and ≤0.01, respectively. Driver mutations were defined as those somatic variants that fulfilled the following criteria: (1) minor allele frequencies (MAF) <0.01 in both ESP6500 (Exome Variant Server, NHLBI GO Exome Sequencing Project) and 1000Genomes databases; (2) calls to those that were nonsynonymous, stopgains, stoplosses, and splicing changes; (3) calls that present in known cancer genes, compiled from the Sanger Cancer Gene Census in COSMIC. Pathways analysis of all genes with the non-silent mutations was performed using the web-accessible program DAVID (Database for Annotation, Visualization, and Integrated Discovery).

### Somatic copy number alterations (SCNA) analysis

For all patients, somatic copy number calls from whole exome data using matched normals were obtained by first deriving segments using Circular Binary Segmentation (CBS) and then deriving log^2^ ratio scores with an in-house tool, exomeCN, which is a modified version of HMMcopy tuned for our data. To delineate recurrent SCNAs detected from the WES data across the 14 patients, copy number segments of samples from the same patients were merged by aligning chromosome segments across samples of the same patient and then assigning the mean of copy number changes to the overlapping chromosomal segments and keeping the copy number changes of those sample-specific segments. Recurrent focal and arm-level somatic SCNAs were then identified using GISTIC2.0 at 95% confidence level. In addition, to prioritize putative important genes from the large size of arm-level SCNAs by leveraging gene expression data, we selected those genes with recurrent SCNAs (log^2^ score cutoff = 0.25) in at least four patients and that were also significantly differentially expressed between DSRCT samples and normal samples (*p* < 0.05 and fold changes > 1.5). In total, 182 DSRCT upregulated genes with copy number gain and 300 downregulated genes with copy number loss were selected (Supplementary Data 3). Pathway analysis of the 482 selected genes was performed using the hypergeometric test.

### Phylogenetic analysis of somatic mutations

Due to low mutation burden in our DSRCT samples, clonal analysis was not applied to phylogenetic reconstruction for avoiding bias. Instead, we simply compared somatic mutations from multiple-site samples of the same patients for phylogenetic reconstruction. Considering enough depth of sequencing is necessary to accurately characterize phylogenetic structure of cancer^32^. Therefore, somatic mutations were validated by capture sequencing from multi-site samples at mean depth of 3700X. The validated mutation profiles of the samples from a patient were then converted into a binary matrix based on the presence and absence of the validated mutations in the samples and the genetic distances between the samples were then calculated using the hamming distance. Phylogenetic relationships between samples of a patient were then inferred using the neighbor-joining algorithm in the phangorn R package. Phylogenetic trees were drawn with relative trunk and branch lengths proportional to the number of shared and distinct mutations of the samples. Ancestors of the tree were germline DNA assuming with no mutations.

### Phylogenetic analysis of SCNAs

The phylogenetic relationship of multi-site biopsies from the same patients was also inferred from the SCNAs. First, the integer SCNA profiles of samples were derived by Sequenza. Second, copy number status of each segment was encoded as follows: -2 (total copy number, tCN = 0), −1 (tCN=1), 0 (tCN=2), 1 (tCN=3), and 2 (tCN > =4). Third, CNtools was applied to convert the encoded segment data of samples from each patient into a site by sample matrix by overlapping segments across samples. Then, similar to phylogenetic analysis of somatic mutations, the genetic distances and phylogenetic relationships between the samples from the same patients were calculated and inferred based on the encoded segment matrix. The phylogenetic tree of a patient was then drawn with relative trunk and branch lengths proportional to the number of shared and distinct SCNA segments of the samples. Ancestors of the tree were germline DNA assuming with no SCNAs.

### RNA Sequencing and gene expression comparison analysis

Total RNA of samples was extracted and libraries made from cDNA using the NuGEN Ovation Ultralow Library System V2 (San Carlos, CA). Libraries from each sample were pooled together in equimolar amounts and sequenced on the Illumina HiSeq 2500 (Illumina, Inc., San Diego, CA). RNA sequencing reads of the samples were mapped to the hg19 reference genome using the STAR aligner. For calculation of gene expression, raw count data of each gene were first obtained using with HTSeq and are normalized by scaling the raw library size using calcNormFactors in edgeR package in R. Then, Voom transformation was applied to normalized counts and a linear model fit to the data for differential expression analysis using the Limma package Significantly deregulated genes between any two groups were selected (*p* ≤ 0.05 and fold change ≥ 1.5). Pathway analyses of differentially expressed genes were performed using Gene Set Enrichment Analysis (GSEA) and the web-accessible program DAVID (Database for Annotation, Visualization, and Integrated Discovery). To detect fusions in our frozen DSRCT samples, we applied an integrative analysis of multiple fusion detection methods. Those fusions that were detected by at least two tools are selected.

### Immune infiltration analysis

Immune infiltration scores of samples across different cancer types were calculated from the gene expression data using ESTIMATE. Immune cell profiles of samples were generated using single-sample Gene Set Enrichment Analysis (ssGSEA) enrichment scores of 29 immune gene signatures.

### Gene sets for evaluating predictions of genes associated with the EWSR1-WT1 fusion

We used several types of benchmark gene sets to comprehensively evaluate our prediction of the EWSR1-WT1 fusion. First, because literature co-citation data have been widely used to infer gene-gene and gene-disease functional associations, we compiled 74 genes cited in at least one paper related to EWSR1-WT1 and 201 genes cited in at least one paper related to DSRCT to evaluate our prediction. All PubMed identification numbers of articles related to EWSR1-WT1 or DSRCT were first downloaded, and then are cross-referenced with the gene citation information from Entrez Gene (ftp://ftp.ncbi.nih.gov/gene/), which is composed of genes and corresponding cited literature. These two benchmark gene sets would help evaluate whether top genes in our prediction include genes known to be associated with EWSR1-WT1 fusion and are associated with the DSRCT oncogenesis. Second, 268 genes whose promoter regions were peak binding sites of WT1 by Chip-seq in the JN-DSRCT1 cell^16^ was used to evaluate our predictions of plausible EWSR1-WT1 direct targets. Third, we also used a set of 328 cancer-related genes compiled from KEGG cancer pathways to evaluate the association between cancer pathways and the fusion. Fourth, to evaluate our predictions of therapeutic targets in the fusion-associated pathways, we collected 194 target genes of 52 compounds that have been tested in clinical trials or used for the treatment of DSRCT (Supplementary Data 8). These target genes were compiled manually from the literature^14,29^ and two public drug databases: DrugBank, and DGIdb^41^.

### Drug testing

JN-DSRCT cell line generously provided from Dr. Kikuchi’s laboratory (Fukuoka University, Fukuoka, Japan), was identified twice per year in MDA characterized cell line core (CCLC) using short tandem repeat (STR) fingerprinting with an AmpFLSTR Identifier kit and tested for mycoplasma contamination using the MycoAlert Detection Kit (Lonza Group Ltd.) according to the manufacturer’s protocol. JN-DSRCT cells were grown in Dulbecco’s Modified Eagle’s Medium (DMEM) (Invitrogen, Cat. #10313) with 10% FBS and 1XPenStrep-Glutamine (Invitrogen, Cat. #15140). The cell proliferation assay was set up by seeding JN-DSRCT cells into a 96-well plate at a density of 5000 cells/well. Cells were incubated overnight at 37 °C, 5% CO_2_. On the following day enzalutamide, alpelisib, and temsirolimus drugs (Selleckchem), serially diluted three-fold in 100% DMSO, were added to cells in the medium at a final concentration of 20 mM (enzalutamide and alpelisib) and 1 mM (temsirolimus). Cells were imaged using the Incucyte (Sartorius) for five and nine days. Phase-contrast images and the confluence of cells untreated or treated with drugs were collected every four hours. Data are presented as mean ± SD of three replicates, a minimum of two independent experiments, or as a percentage of untreated cells (control DMSO) corresponding to the percentage inhibition of proliferation. IC50 values were calculated by the sigmoidal dose-response curve-fit using Prism GraphPad 8.0.

### ARID1A knockout

The sgRNA sequences are from the Avana whole-genome library provided by the Broad Institute. The sgRNAs were amplified and cloned into a custom backbone (LentiCRISPR-E-HK14-10xCS) developed by the TRACTION group here at MDACC that has puromycin resistance for selection. The sgRNA has been validated and sequence confirmed. Three against *ARID1A* were chosed based on dependency scores obtained across the Achilles screening platform of over 600 cell lines. The screen’s dependency calls were converted to Bayes Factors (BF) at the guide level. Then BF values in the range 0–5 were converted to a value of 0.5. BF values greater than 5 were converted to a value of 1. Any BF value <0 was converted to 0. After the conversion of BF values for each guide in each of the 625 cell lines, a sum of dependency value was calculated. The *ARID1A* sgRNA sequences were as follows: KO#1 GGGCTACCAGGGCTACCCCG; KO#2 GTGTGTATCTGTCCTCCGGA; KO#3 TCCTGCAGCTCCTTTCCCAG. The non-targeting control against luciferase sgRNA was: ACAACTTTACCGACCGCGCC. Infectious viral particles were produced using helper plasmids psPAX2 and pMD2G that were obtained through Addgene. HEK293T cells (ATCC accession number CRL-1573) were cultured in DMEM containing 10% FBS (Gibco), (100 U/mL) 1% penicillin–streptomycin (Gibco) and transfected using the Polyethylenimine (PEI) method (1). After 72 h of transfection, supernatant containing virus was collected and centrifuged at 3000 rpm for 10 min to remove dead cell debris. High-titer preps were obtained by ultracentrifugation of supernatant containing virus at 24,000 RPM for 2.5 h at 4 °C. JN-DSRCT cell lines were then infected with the concentrated viral preps in drop-wise fashion, and 1 μg/ml of polybrene was added to the culture medium to improve the infection efficiency of cells prior to the addition of virus. After 24 h of incubation with the virus, 1 μg/ml puromycin was added to JN-DRSCT cells to select for successful integration of puromycin resistant vector containing sgRNA. The puromycin-supplemented medium was replaced at every passage throughout culturing until the knockout cells were selected. Parent cell line without knockout was used as a negative control and was expected to die after 1–2 days of culturing with equal concentrations of puromycin. After one week of selection, cell lysates were prepared for immunoblotting analysis. RIPA buffer supplemented with protease inhibitor was used to lyse the cells on ice. Protein lysates were mixed with an equal volume of Laemmli Sample Buffer (Bio-Rad), heat denatured (95 °C, 10 min) with β-mercaptoethanol (β-ME; Sigma-Aldrich), loaded in precast SDS/PAGE gels (Bio-Rad), transferred to nitrocellulose membranes, and probed with specific primary antibodies overnight at 4 °C. The following day, they were probed with secondary anti-mouse or anti-rabbit IgG conjugated horseradish peroxidase antibody (Cell Signaling Technologies, 1:2000), and chemiluminescence was detected by film exposure. The following primary antibodies were used: ARID1A/BAF250A (1:1000, Cell Signaling Technologies, D2A8U) and β-actin (1:1000, Cell Signaling Technologies).

### Reporting summary

Further information on research design is available in the Nature Research Reporting Summary linked to this article.

## Supplementary information

Supplementary Material

Dataset 1

Dataset 2

Dataset 3

Dataset 4

Dataset 5

Dataset 6

Dataset 7

Dataset 8

Dataset 9

REPORTING SUMMARY

## Data Availability

The exome and RNA sequencing data were deposited at the European Genome-phenome Archive under the study accession number: EGAS00001004575.
