# Supplementary material for: Multi-site desmoplastic small round cell tumors are genetically related and immune-cold
Source: NPJ Precis Oncol. 2022 Apr 4;6:21. doi: 10.1038/s41698-022-00257-9 (PMC8980094; doi:10.1038/s41698-022-00257-9)
Supplement: Supplementary file 1 — Supplementary Material [file 41698_2022_257_MOESM1_ESM.pdf]

Supplementary Figures

Supplementary Figure 1: Mutation landscape of genes recurrently mutated in at least two patients. Genes are shown according to specimens, so mutations in different specimens from the same patient were shown multiple times. The landscape of recurrently mutated genes for at the patient level is found in Figure 1B

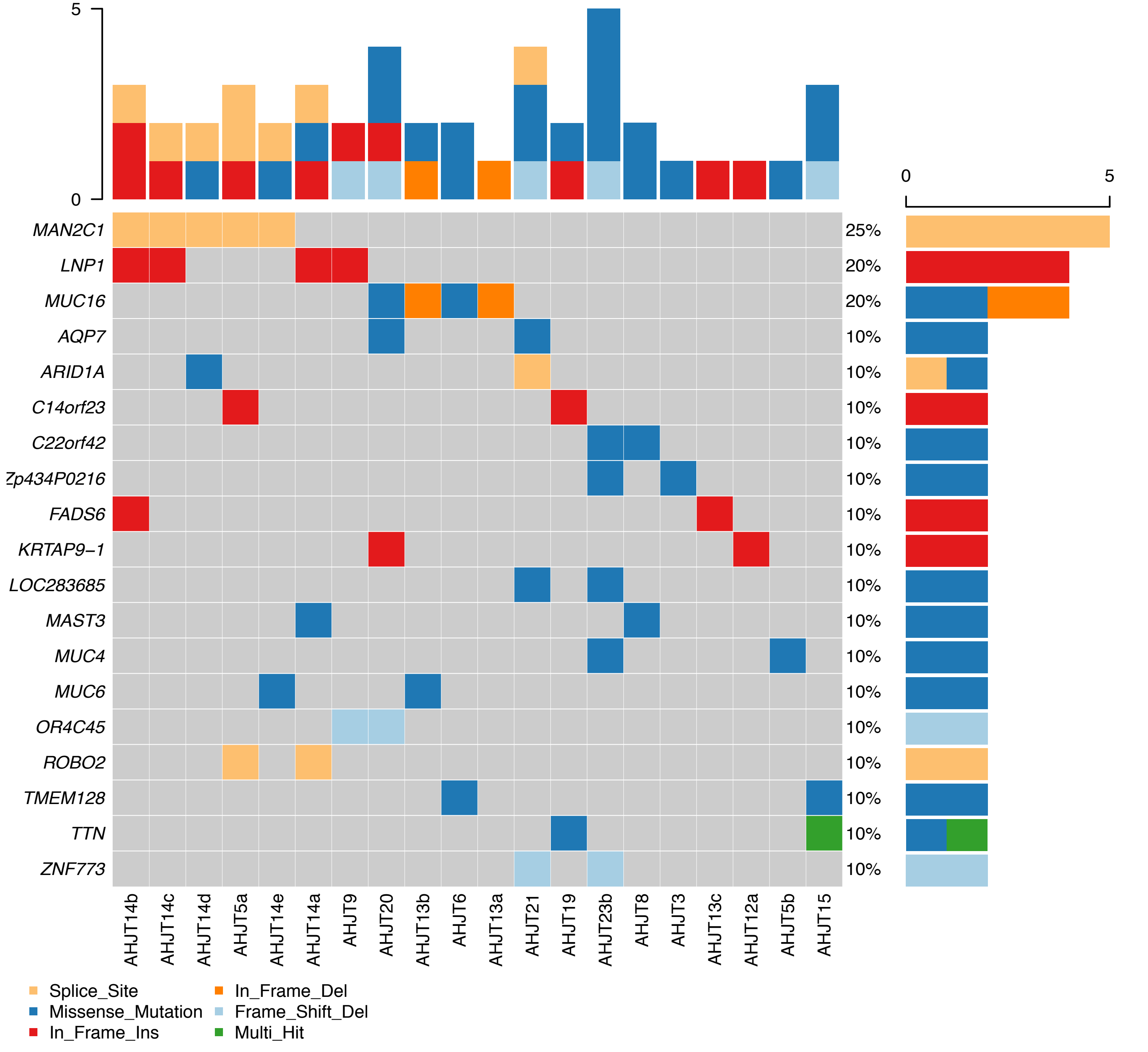

Supplementary Figure 2: IGV copy number profiles of all 23 samples. Profiles of samples from the same patients were clustered together.

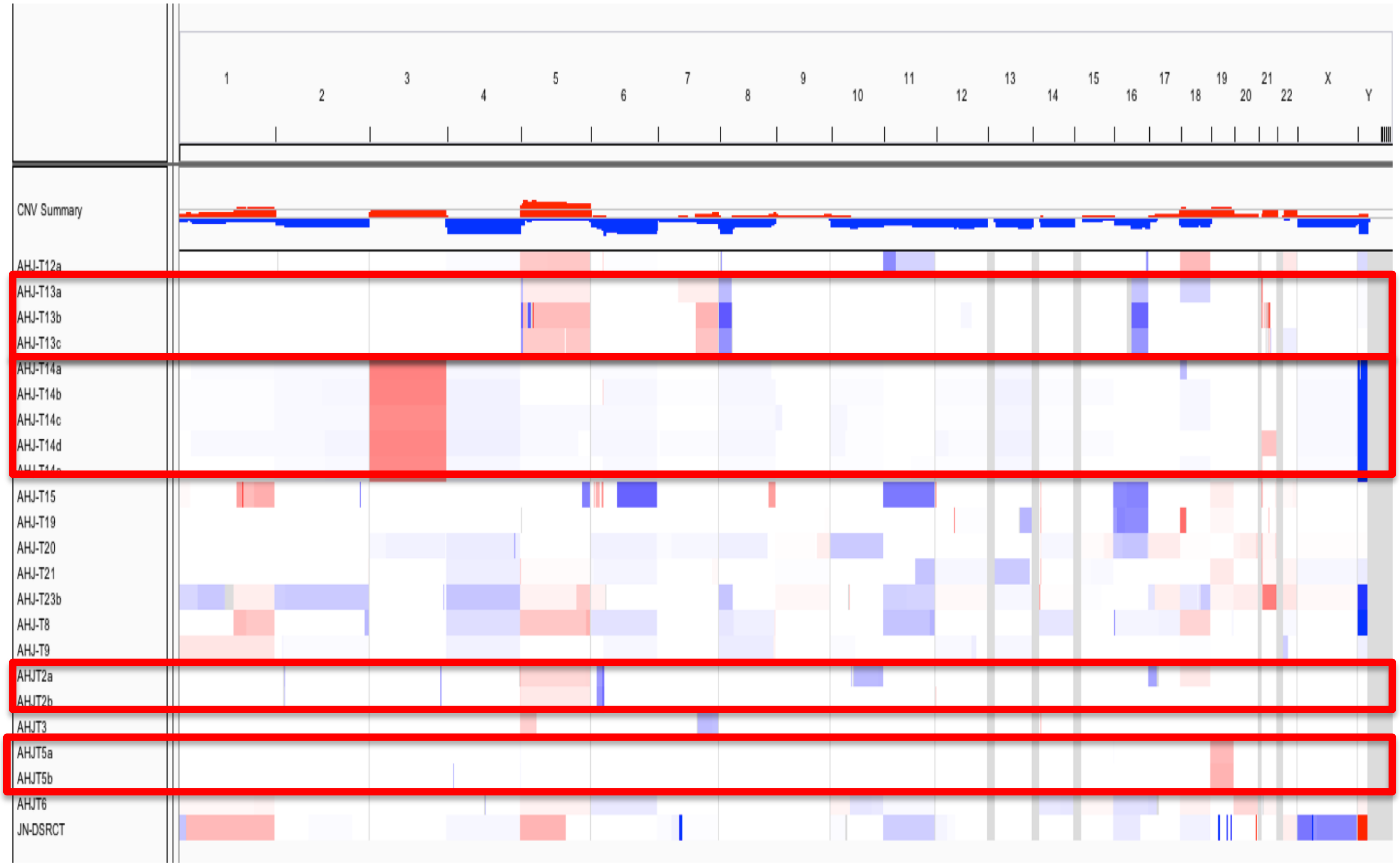

Supplementary Figure 3: Copy number profiles of samples of patient AHJT2

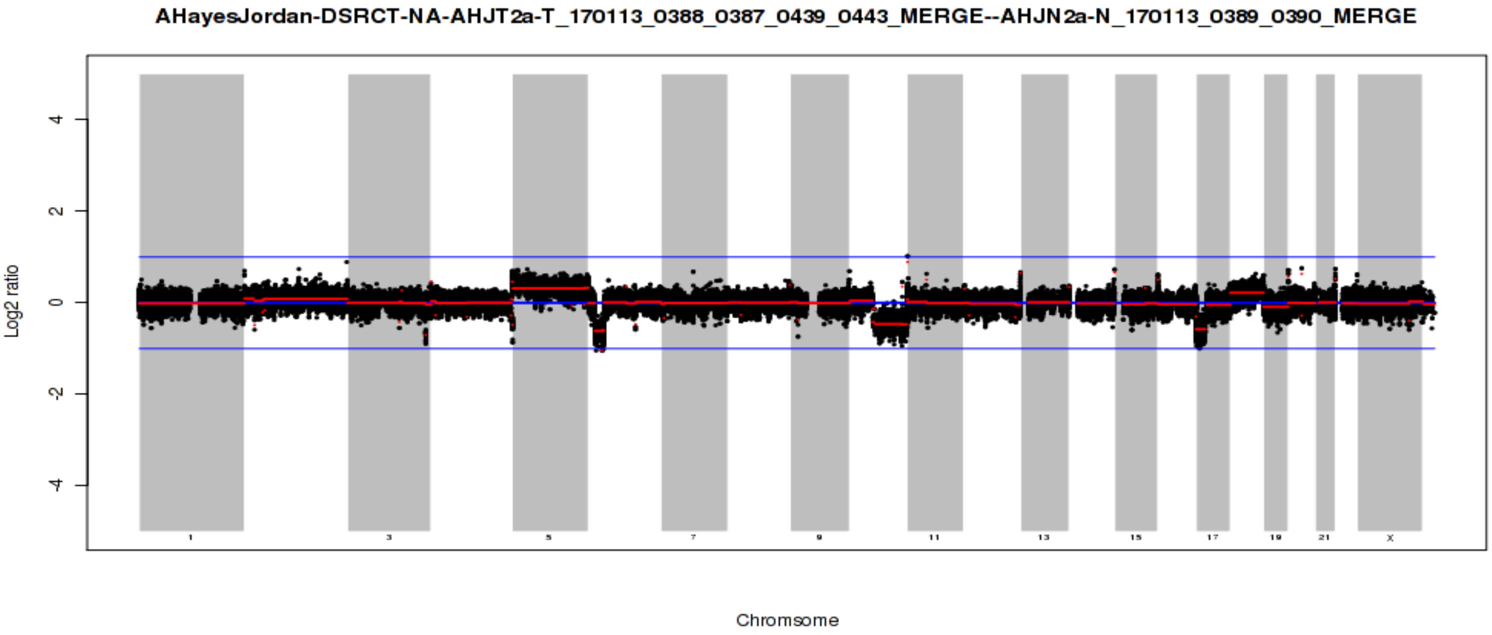

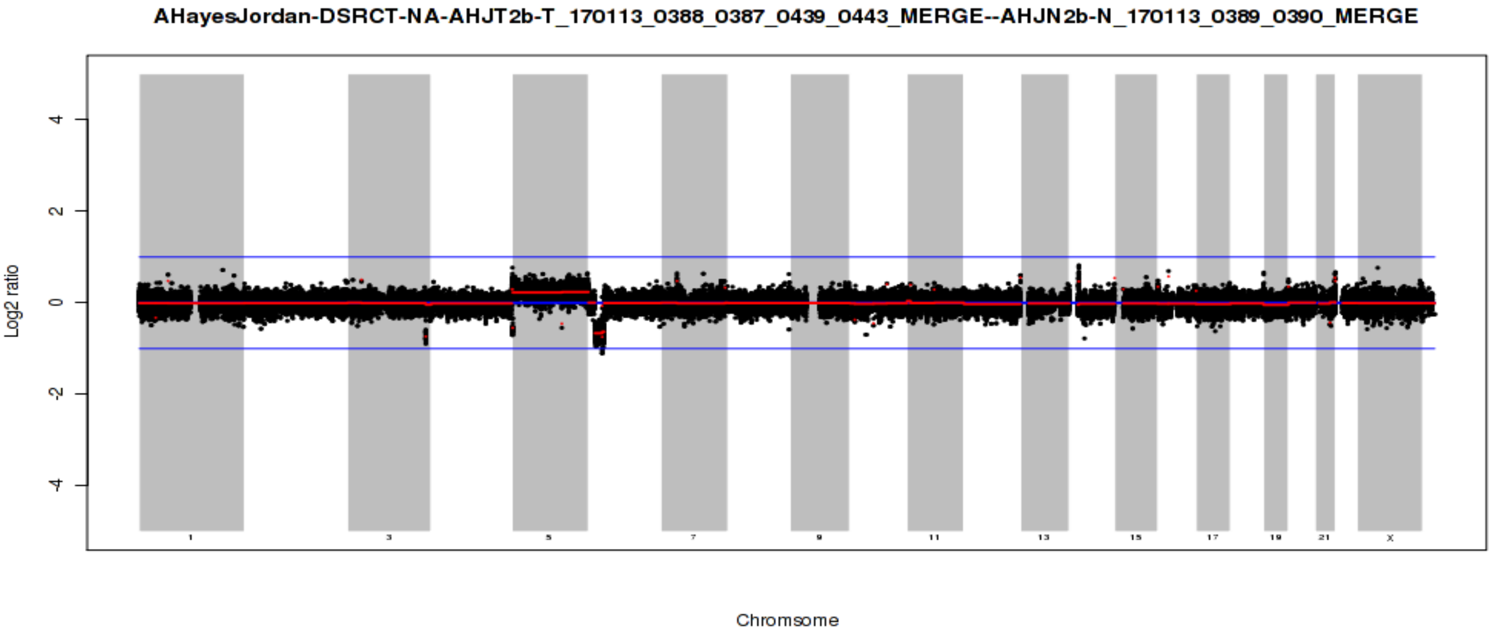

Supplementary Figure 4: Copy number profiles of samples of patient AHJT5

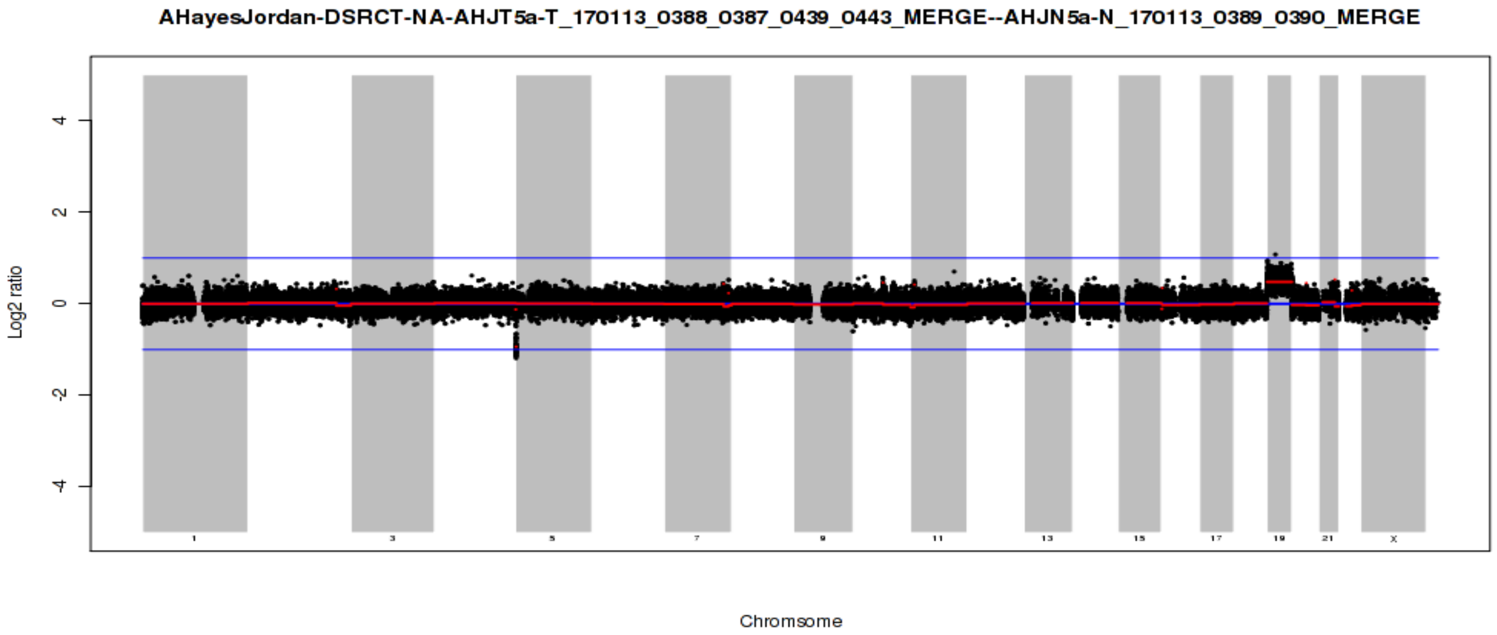

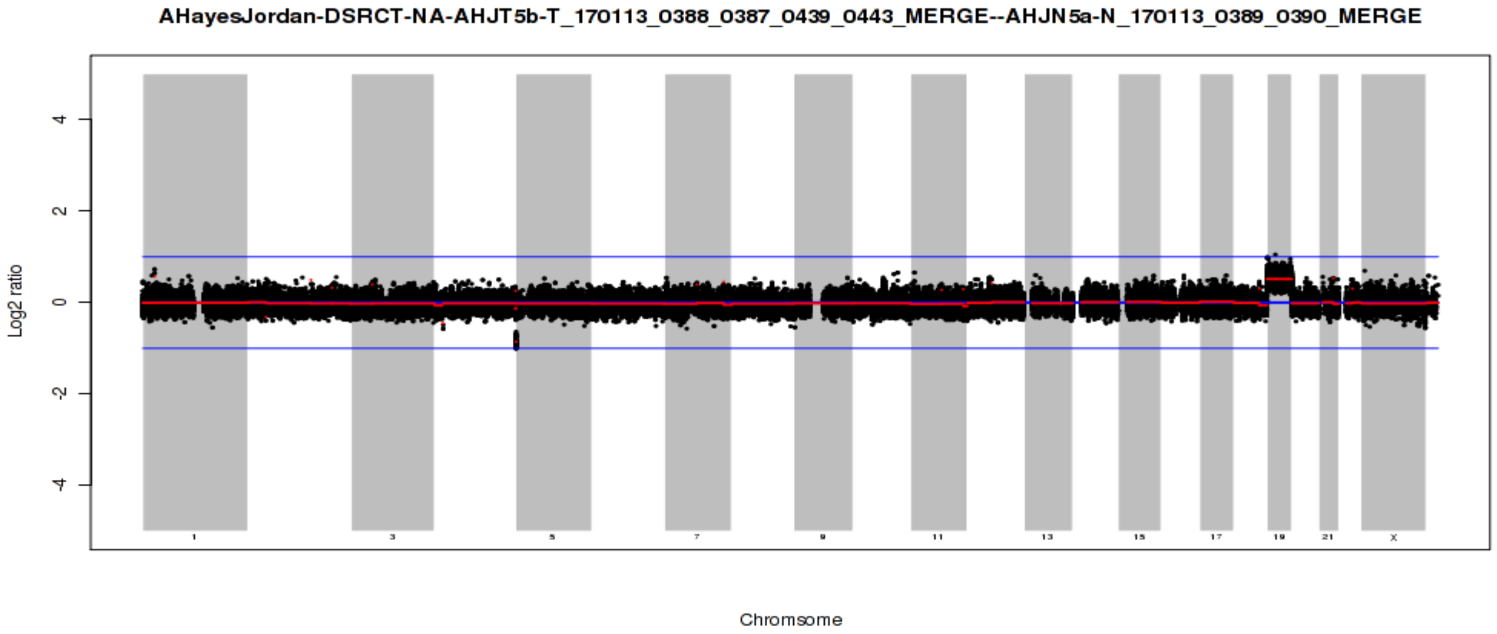

Supplementary Figure 5: Copy number profiles of samples of patient AHJT13

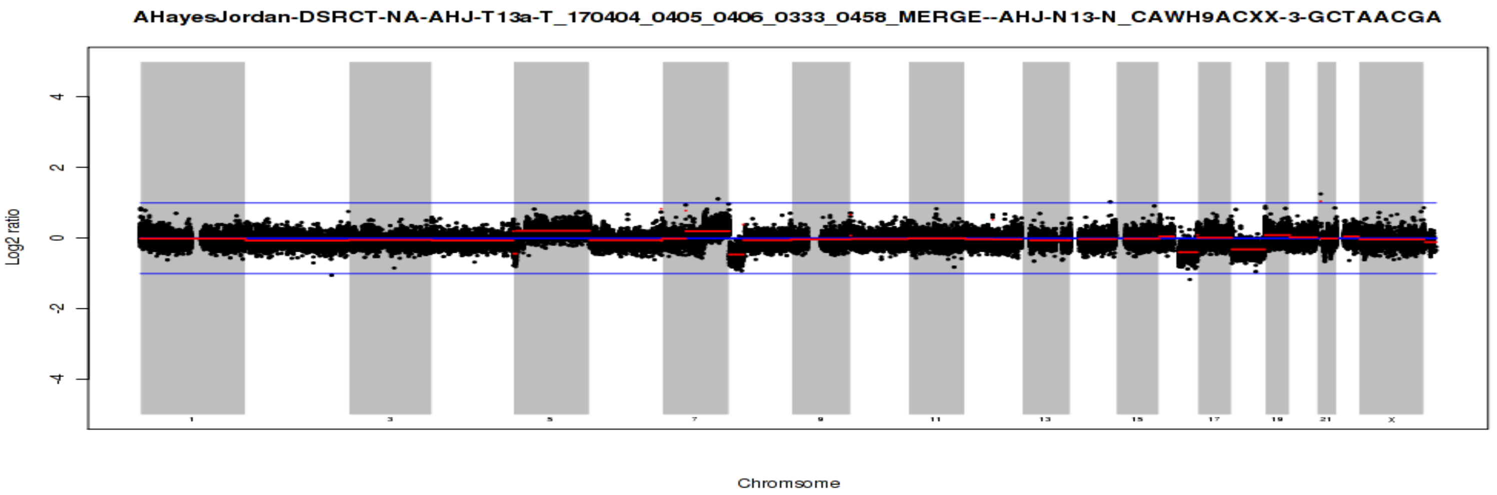

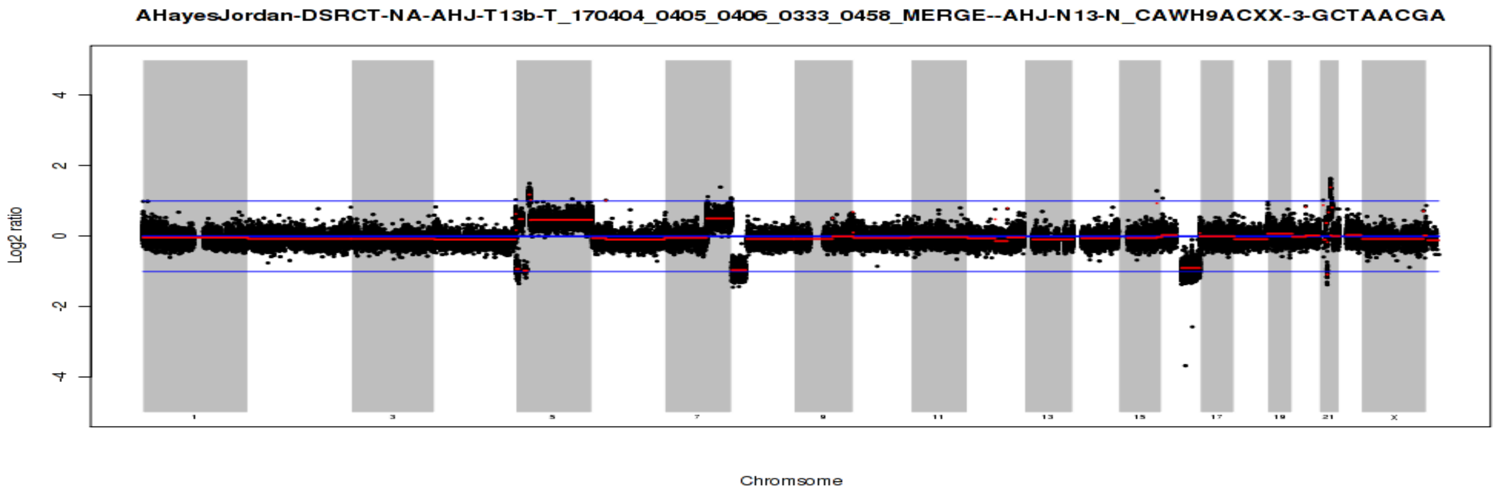

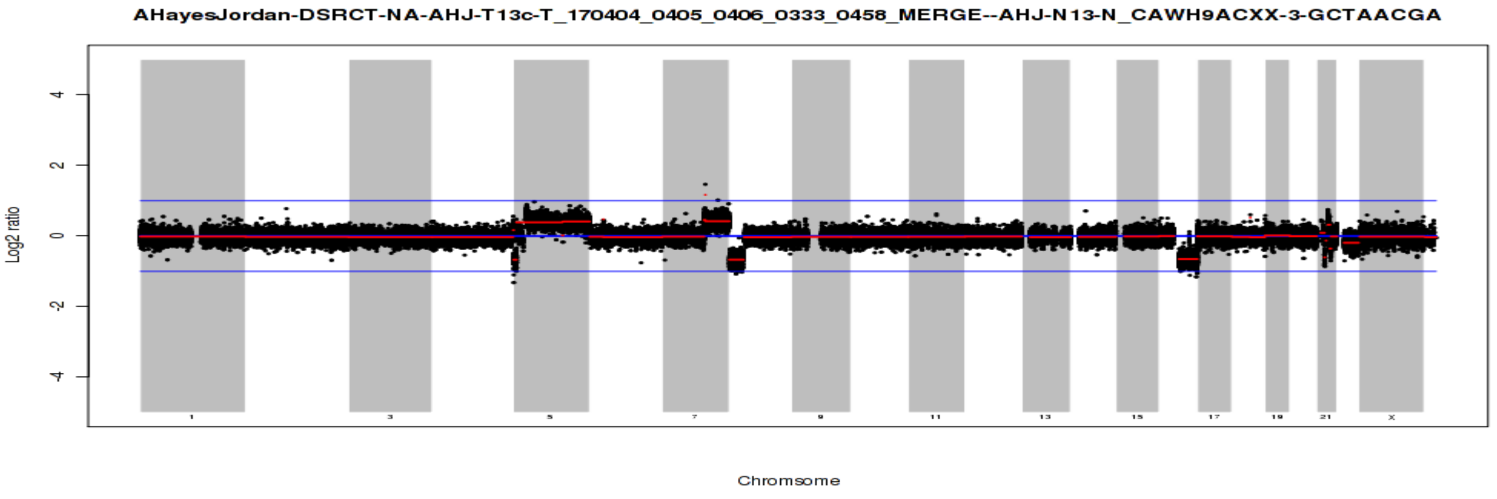

Supplementary Figure 6: Copy number profiles of samples of patient AHJ-T14

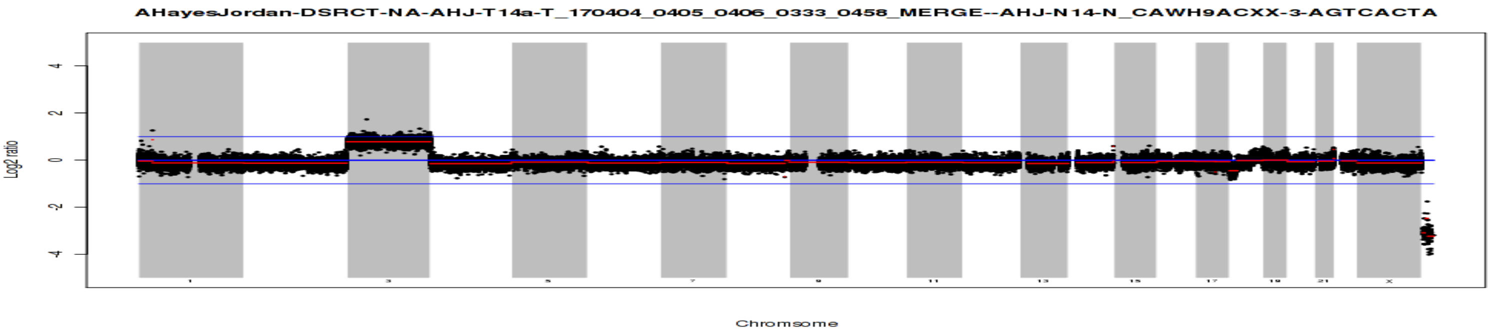

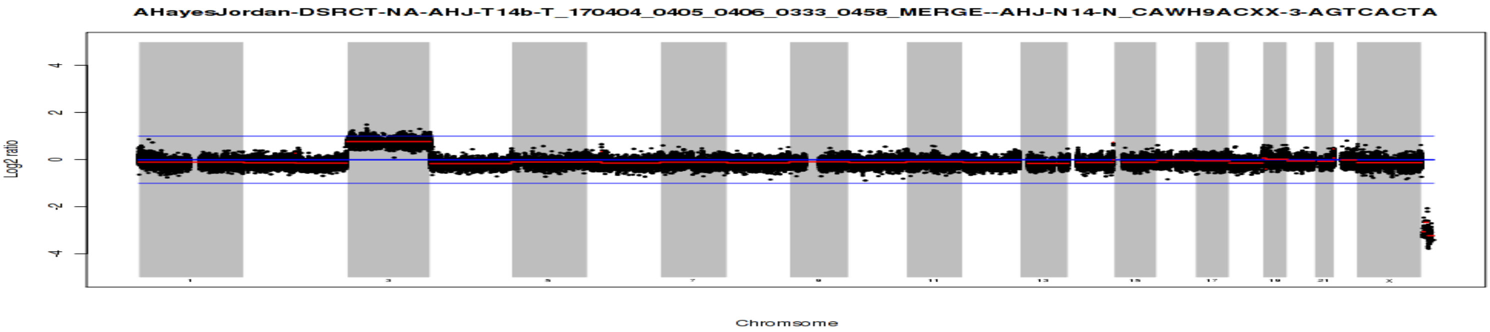

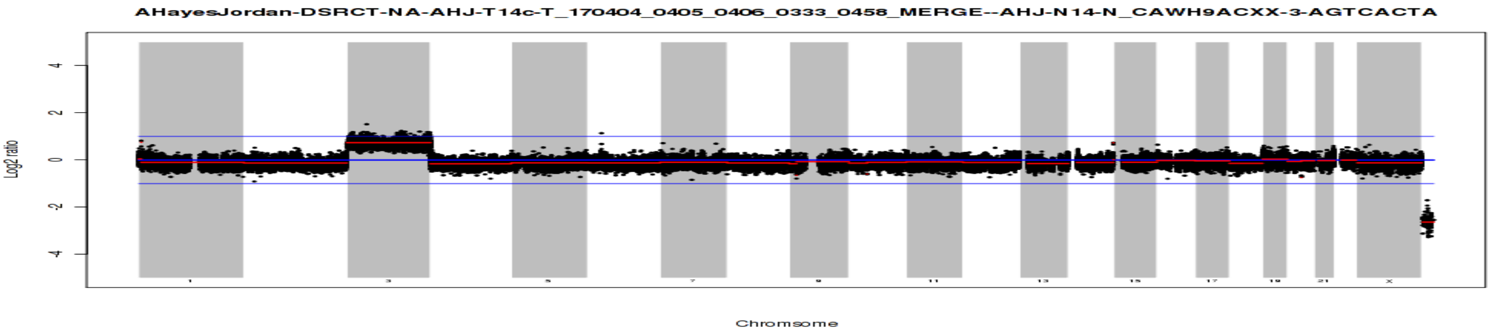

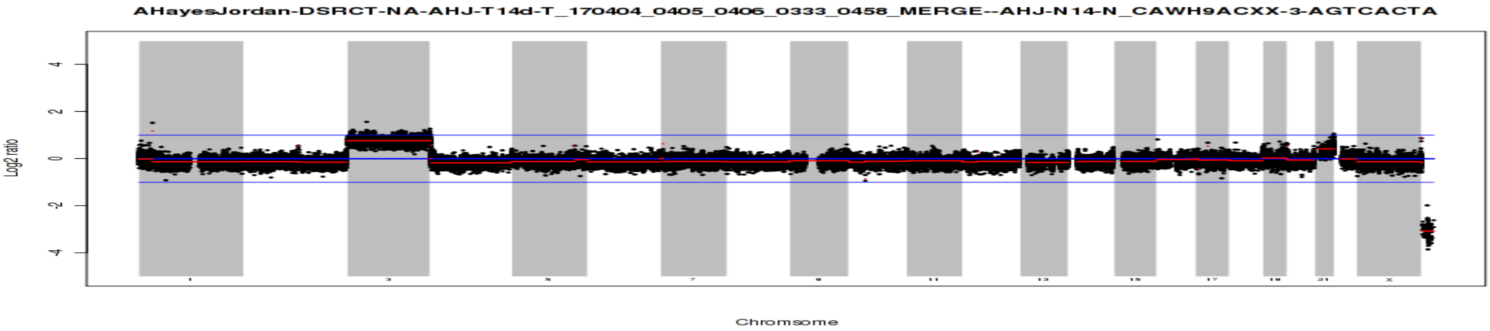

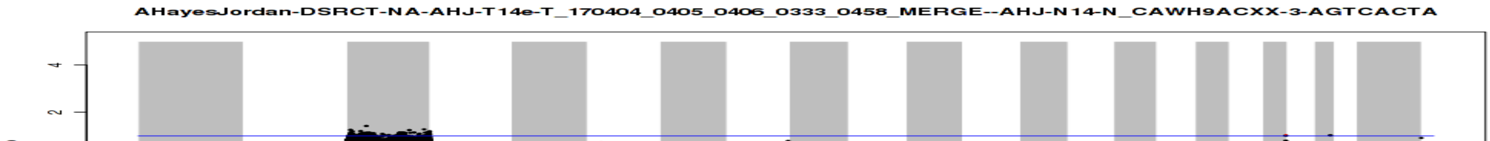

Supplementary Figure 7: Correlation analysis of SCNA profiles between all the samples. Heat map represents correlation coefficients.

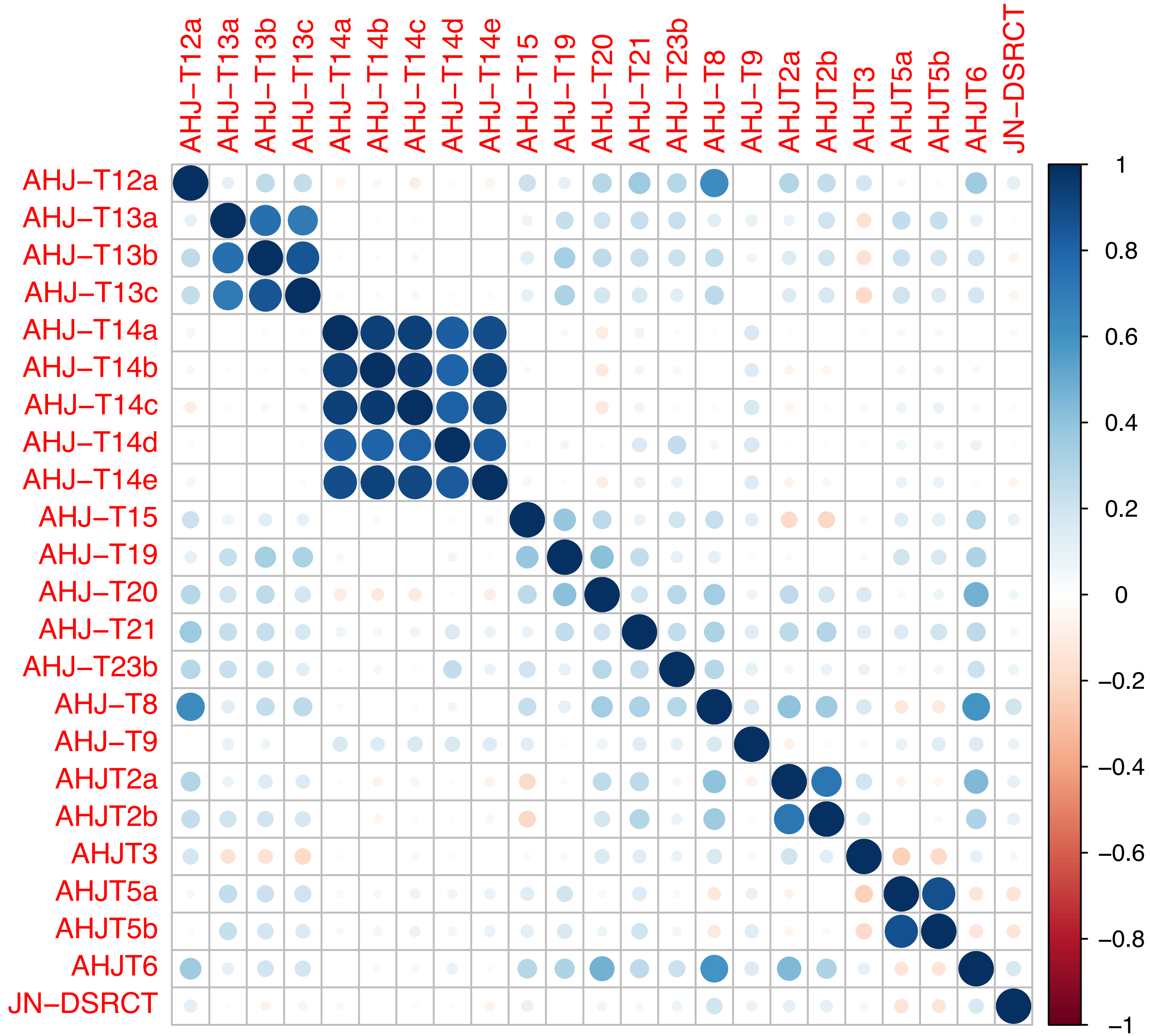

Supplementary Figure 8: SCNA phylogenetic trees of patient AHJT-2T, 5T, 13T, and 14T. Some arm-level or whole chromosomes SCNAs are mapped to the trunks and branches as indicated. Aberrations are annotated as follows: (--) homozygous deletions, (-) losses, (+) gains, (++) amplicons.

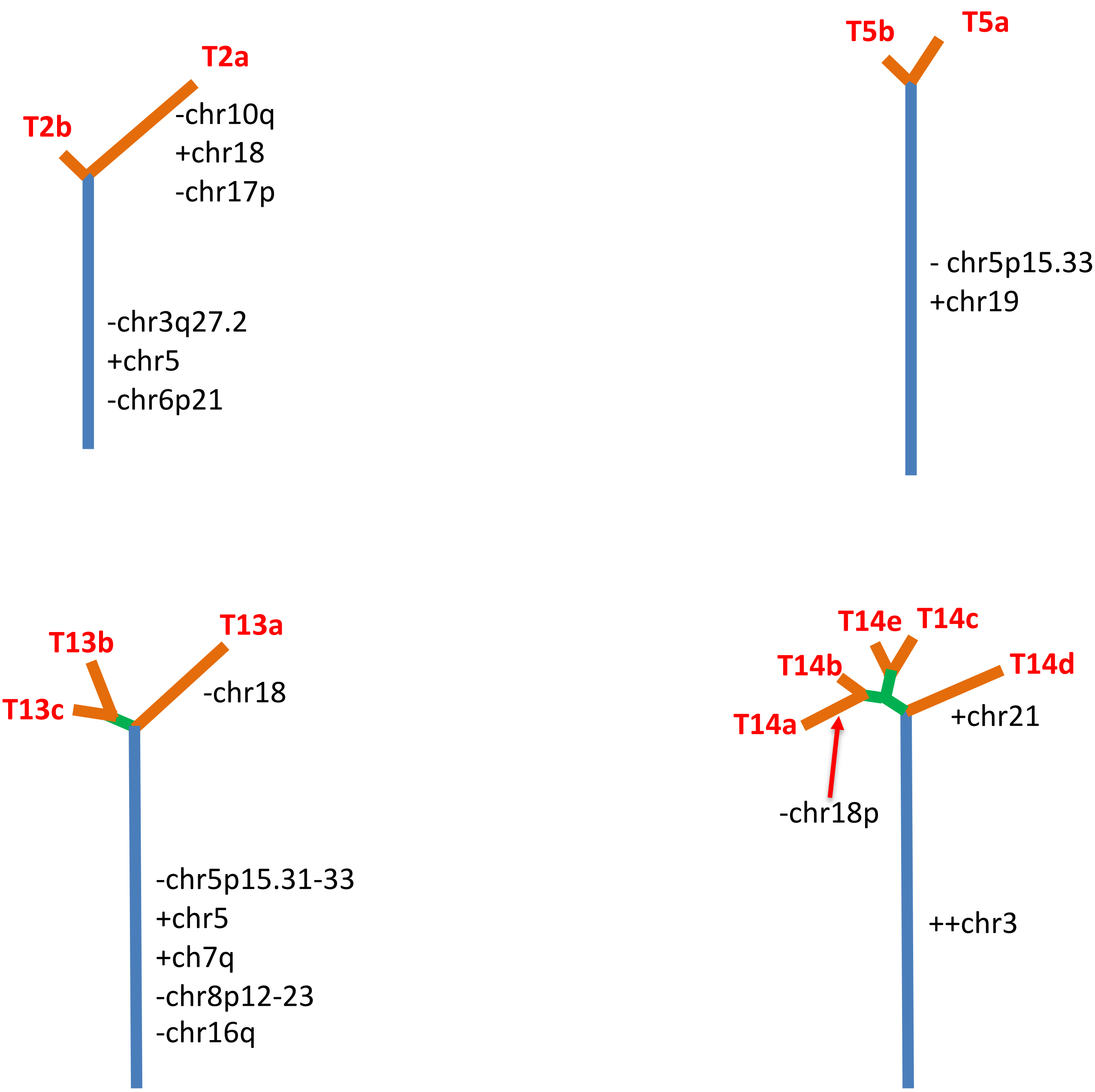

Supplementary Figure 9: Volcano plot of gene expression comparison between DSRCT and adjacent normal tissues

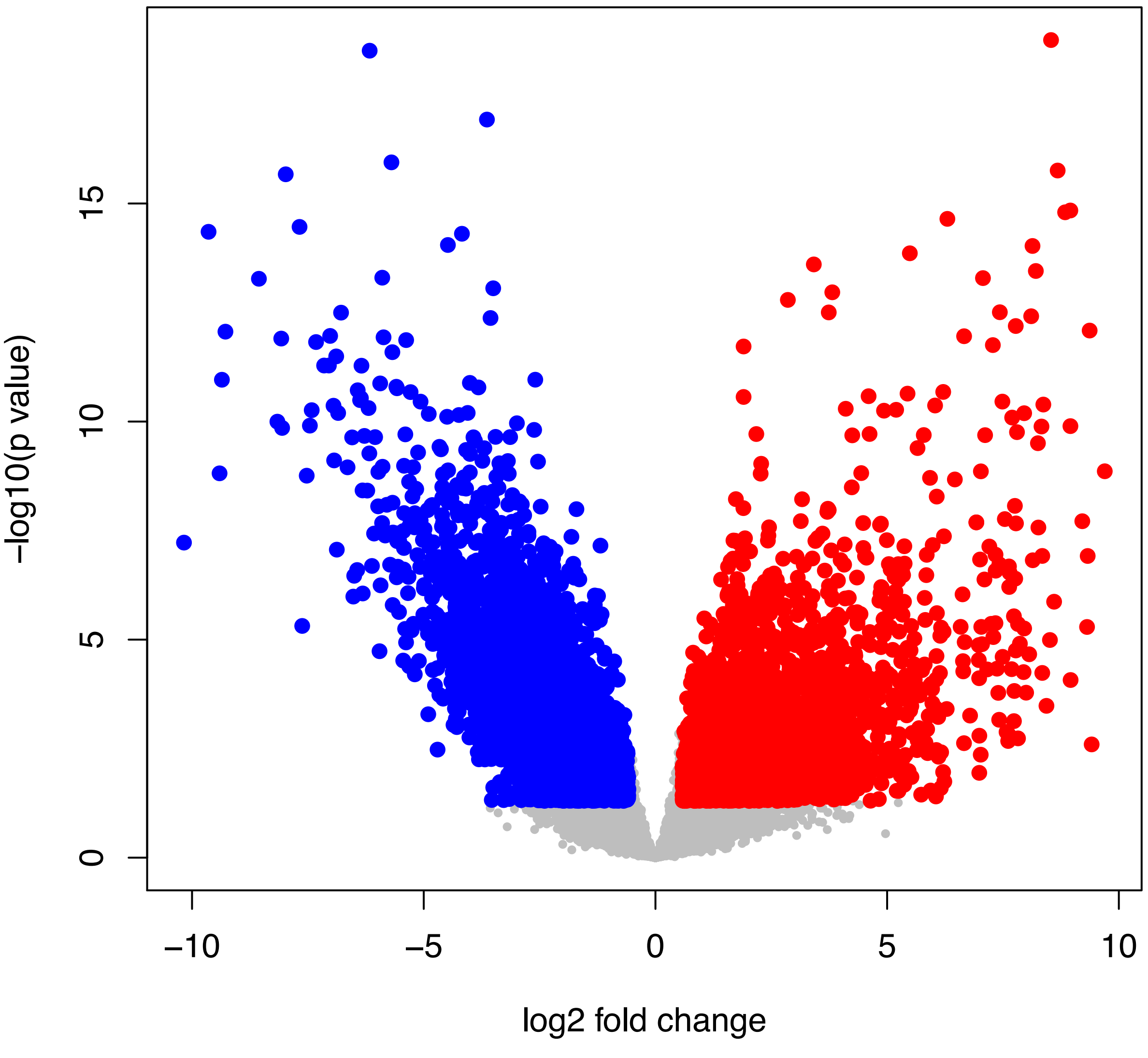

Supplementary Figure 10. The flowcharts of network integrative analyses. A. Identification of pathways that are associated with the EWS-WT1 in DSRCT. **B.** Identification of potential therapeutic genes associated with the EWS-WT1 in DSRCT.

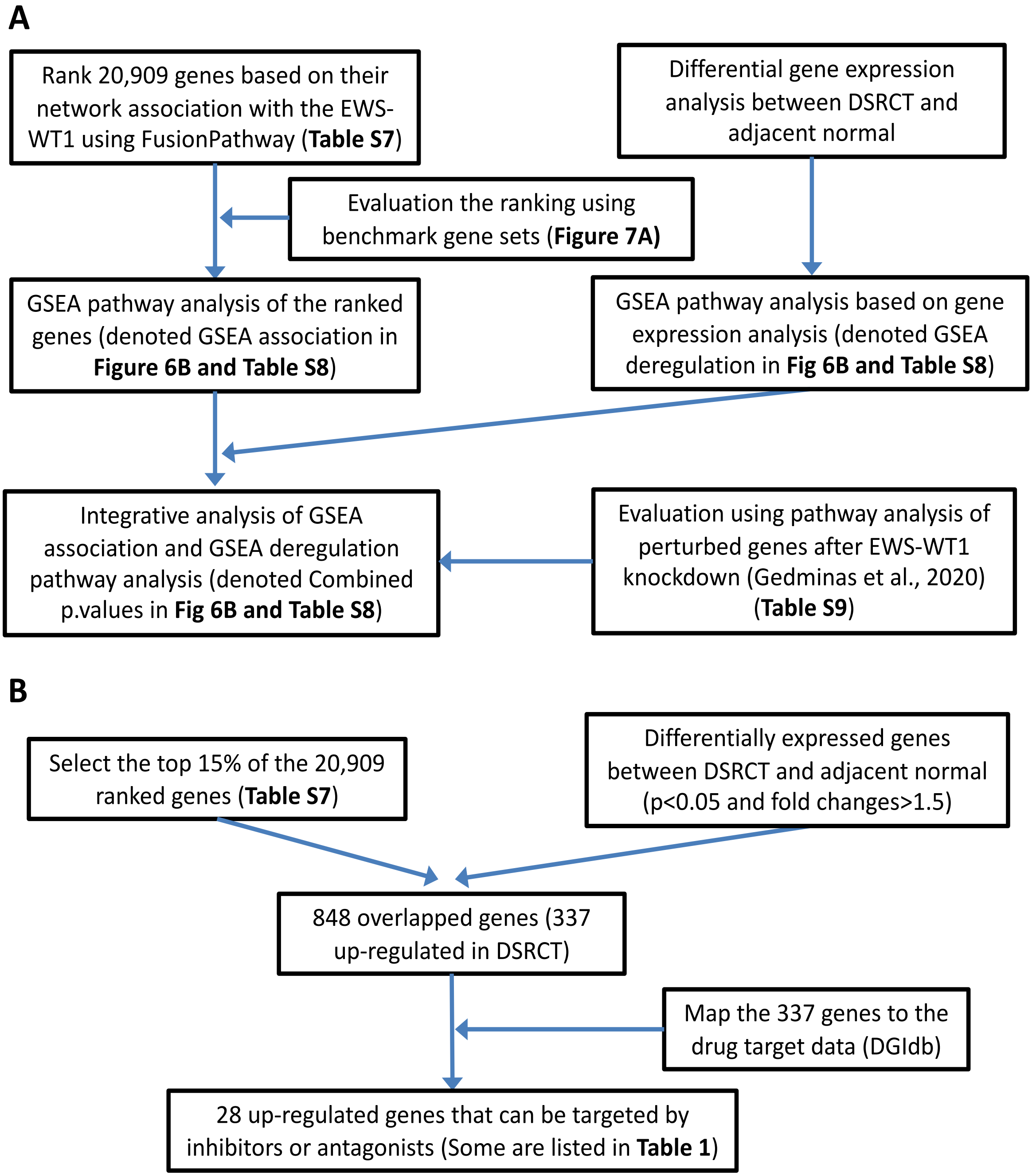

Supplementary Figure 11: Prediction evaluation of genes associated with EWS-WT1 using 5 benchmark gene sets. *p*-values of Wilcoxon test quantifying the performance of our prediction. The vertical red dotted lines in the bar plot represent P= 0.05.

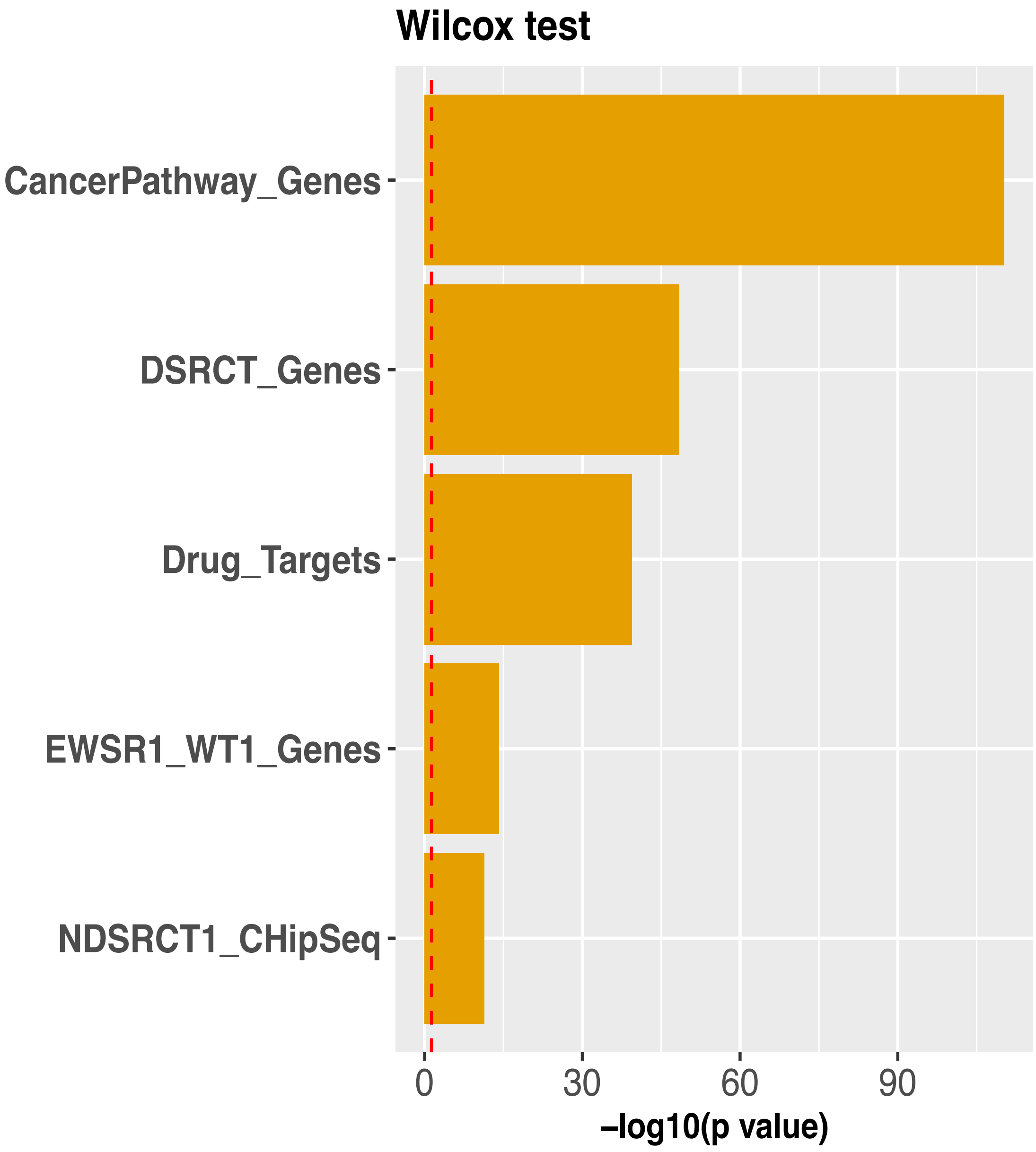

Supplementary Figure 12: Bar plot of pathways shown in Figure 6B. Similar to Figure 6B, the bar plot also shows the statistical significance of a given pathway in GSEA association analysis, but the statistical significance of GSEA deregulation analysis was obtained by gene expression analysis between DSRCT and other types of sarcoma

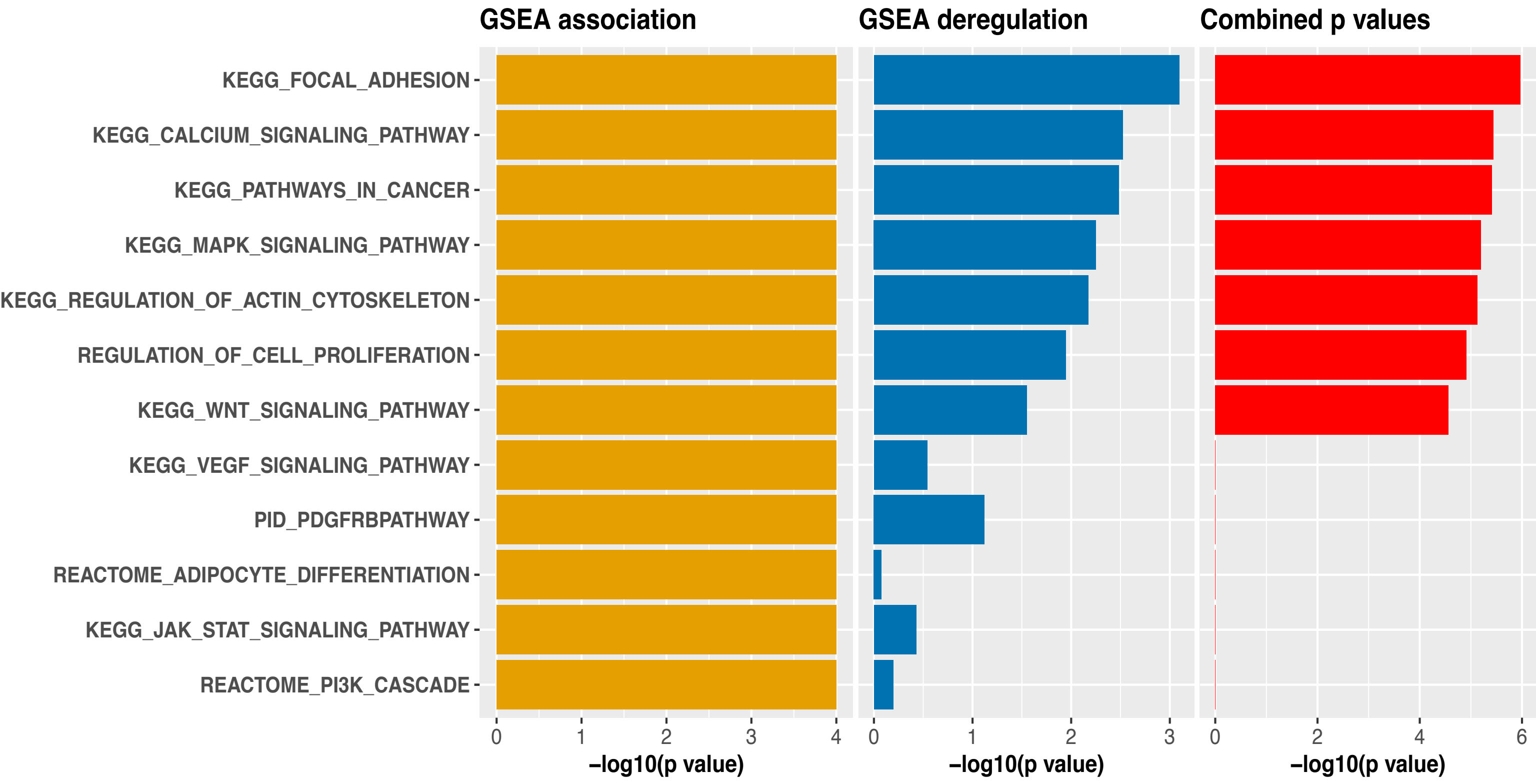

Supplementary Tables

Supplementary Table 1: Patient table

Patient (number of regions sequenced)	Sample_ID	Matched Normal	WT1_fusion	DSRC_Patient	DSRCT_TissueSource	Sex	WES	RNAseq
AHJT2 (2)	AHJT2a	Y	Y	AHJ2	-	Male	Y	Y
	AHJT2b	Y	Y	AHJ2	-	Male	Y	Y
AHJT3 (1)	AHJT3	Y	Y	AHJ3	-	Male	Y	Y
AHJT5 (2)	AHJT5a	Y	Y	AHJ5	-	Male	Y	Y
	AHJT5b	Y	Y	AHJ5	-	Male	Y	Y
AHJT6 (1)	AHJT6	Y	Y	AHJ6	-	Male	Y	Y
AHJT8 (1)	AHJ-T8	Y	Y	AHJ8	-	Male	Y	Y
AHJT9 (1)	AHJ-T9	Y	Y	AHJ9	-	Male	Y	Y
AHJT12 (1)	AHJ-T12a	Y	Y	AHJ12	SCN (Colon)	Male	Y	Y
AHJT13 (3)	AHJ-T13a	Y	Y	AHJ13	Omentum	Male	Y	Y
	AHJ-T13b	Y	Y	AHJ13	RUQ peritoneum	Male	Y	Y
	AHJ-T13c	Y	Y	AHJ13	Pelvic Peritoneum	Male	Y	Y
AHJT14 (5)	AHJ-T14a	Y	Y	AHJ14	Retro peritoneum	Male	Y	Y
	AHJ-T14b	Y	Y	AHJ14	Diaphragm	Male	Y	Y
	AHJ-T14c	Y	Y	AHJ14	Omentum	Male	Y	Y
	AHJ-T14d	Y	Y	AHJ14	Colon	Male	Y	Y
	AHJ-T14e	Y	Y	AHJ14	Pelvis	Male	Y	Y
AHJT15 (1)	AHJ-T15	Y	Y	AHJ15	Peritoneum	Male	Y	Y
AHJT19 (1)	AHJ-T19	Y	Y	AHJ19	-	Male	Y	Y
AHJT20 (1)	AHJ-T20	Y	Y	AHJ20	Peritoneum	Male	Y	Y
AHJT21 (1)	AHJ-T21	Y	Y	AHJ21	Omentum	Male	Y	Y
AHJT23 (1)	AHJ-T23b	Y	Y	AHJ23	Omentum	Male	Y	Y
JN-DSRCT (NA)	JN-DSRCT	N	Y	JN-DSRC	DSRCT cell	Male	Y	Y
**Normal tissues with RNA-sequencing data**								
	AHJ-N8	Y		AHJ8	-	Male	Y	Y
	AHJN6	Y		AHJ6	omentum	Male	Y	Y
**TOTAL NUMBER OF PATIEN**	14							

Supplementary Table 2: Abbreviations of the tumour types in Figure 4A

ACC	adrenocortical carcinoma
BLCA	bladder urothelial carcinoma
BRCA	breast invasive carcinoma
CESC	cervical squamous cell carcinoma and endocervical adenocarcinoma
CHOL	cholangiocarcinoma
COAD	colon adenocarcinoma
ESCA	esophageal carcinoma
GBM	glioblastoma multiforme
HNSC	head and neck squamous cell carcinoma
KICH	kidney chromophobe
KIRC	renal clear cell carcinoma
KIRP	kidney renal papillary cell carcinoma
LGG	low-grade glioma
LIHC	liver hepatocellular carcinoma
LUAD	lung adenocarcinoma
LUSC	lung squamous cell carcinoma
MDACC.DSRCT	desmoplastic small round cell tumor
MESO	mesothelioma
OV	ovarian serous cystadenocarcinoma
PAAD	pancreatic adenocarcinoma
PCPG	pheochromocytoma and paraganglioma
PRAD	prostate adenocarcinoma
READ	rectum adenocarcinoma
SARC.DDLPS	dedifferentiated liposarcoma
SARC.LMS	leiomyosarcoma
SARC.MFS	myxofibrosarcoma
SARC.MPNST	malignant peripheral nerve sheath tumors
SARC.UPS	undifferentiated pleomorphic sarcoma
SARC.SS	synovial sarcoma
SKCM	skin cutaneous melanoma
STAD	stomach adenocarcinoma
STES	stomach and esophageal carcinoma
TGCT	testicular germ cell tumors
THCA	thyroid carcinoma
UCEC	uterine corpus endometrial carcinoma
UCS	uterine carcinosarcoma
UVM	uveal melanoma

Supplementary References

References for the tools and other methods used:

1. Li, H. Aligning sequence reads, clone sequences and assembly contigs with BWA-MEM. 2013.
2. Cibulskis, K., et al., Sensitive detection of somatic point mutations in impure and heterogeneous cancer samples. Nat Biotechnol, 2013. 31(3): p. 213-9.
3. Ye, K., et al., Pindel: a pattern growth approach to detect break points of large deletions and medium sized insertions from paired-end short reads. Bioinformatics, 2009. 25(21): p. 2865-71.
4. Genomes Project, C., et al., A global reference for human genetic variation. Nature, 2015. 526(7571): p. 68-74.
5. Forbes, S.A., et al., COSMIC: mining complete cancer genomes in the Catalogue of Somatic Mutations in Cancer. Nucleic Acids Res, 2011. 39(Database issue): p. D945-50.
6. Huang da, W., B.T. Sherman, and R.A. Lempicki, Systematic and integrative analysis of large gene lists using DAVID bioinformatics resources. Nat Protoc, 2009. 4(1): p. 44-57.
7. Olshen, A.B., et al., Circular binary segmentation for the analysis of array-based DNA copy number data. Biostatistics, 2004. 5(4): p. 557-72.
8. Ha, G., et al., Integrative analysis of genome-wide loss of heterozygosity and monoallelic expression at nucleotide resolution reveals disrupted pathways in triple-negative breast cancer. Genome Res, 2012. 22(10): p. 1995-2007.

9. Mermel, C.H., et al., GISTIC2.0 facilitates sensitive and confident localization of the targets of focal somatic copy-number alteration in human cancers. Genome Biol, 2011. 12(4): p. R41.
10. Schliep, K.P., phangorn: phylogenetic analysis in R. Bioinformatics, 2011. 27(4): p. 592-3.
11. Favero, F., et al., Sequenza: allele-specific copy number and mutation profiles from tumor sequencing data. Ann Oncol, 2015. 26(1): p. 64-70.
12. Dobin, A., et al., STAR: ultrafast universal RNA-seq aligner. Bioinformatics, 2013. 29(1): p. 15-21.
13. Anders, S., P.T. Pyl, and W. Huber, HTSeq--a Python framework to work with high-throughput sequencing data. Bioinformatics, 2015. 31(2): p. 166-9.
14. Robinson, M.D., D.J. McCarthy, and G.K. Smyth, *edgeR: a Bioconductor package for differential expression analysis of digital gene expression data.* Bioinformatics, 2010. **26**(1): p. 139-40.
15. Law, C.W., et al., voom: Precision weights unlock linear model analysis tools for RNA-seq read counts. Genome Biol, 2014. 15(2): p. R29.
16. Smyth, G.K., limma: Linear Models for Microarray Data, in Bioinformatics and Computational Biology Solutions Using R and Bioconductor, R. Gentleman, et al., Editors. 2005, Springer New York: New York, NY. p. 397-420.
17. Subramanian, A., et al., Gene set enrichment analysis: a knowledge-based approach for interpreting genome-wide expression profiles. Proc Natl Acad Sci U S A, 2005. 102(43): p. 15545-50.

18. Wang, K., et al., MapSplice: accurate mapping of RNA-seq reads for splice junction discovery. Nucleic Acids Res, 2010. 38(18): p. e178.
19. Kim, D. and S.L. Salzberg, TopHat-Fusion: an algorithm for discovery of novel fusion transcripts. Genome Biol, 2011. 12(8): p. R72.
20. Ge, H., et al., FusionMap: detecting fusion genes from next-generation sequencing data at base-pair resolution. Bioinformatics, 2011. 27(14): p. 1922-8.
21. Wu, C.C., et al., Prediction of human functional genetic networks from heterogeneous data using RVM-based ensemble learning. Bioinformatics, 2010. 26(6): p. 807-13.
22. Knox, C., et al., DrugBank 3.0: a comprehensive resource for 'omics' research on drugs. Nucleic Acids Res, 2011. 39(Database issue): p. D1035-41.
23. Beird, H. C. *et al.* Genomic profiling of dedifferentiated liposarcoma compared to matched well-differentiated liposarcoma reveals higher genomic complexity and a common origin. Cold Spring Harb Mol Case Stud, 2018. 4, doi:10.1101/mcs.a002386.
24. Wu, C. C. *et al.* Immuno-genomic landscape of osteosarcoma. *Nat Commun* **11**, 1008, doi:10.1038/s41467-020-14646-w (2020).
