# Supplementary material for: Multi-site desmoplastic small round cell tumors are genetically related and immune-cold
Source: NPJ Precis Oncol. 2022 Apr 4;6:21. doi: 10.1038/s41698-022-00257-9 (PMC8980094; doi:10.1038/s41698-022-00257-9)
Supplement: Supplementary file 11 — REPORTING SUMMARY [file 41698_2022_257_MOESM11_ESM.pdf]

nature portfolio

Corresponding author(s): Andrew Futreal, Andrea Hayes-Jordan

Last updated by author(s): Dec 11, 2021

Reporting Summary

Nature Portfolio wishes to improve the reproducibility of the work that we publish. This form provides structure for consistency and transparency in reporting. For further information on Nature Portfolio policies, see our Editorial Policies and the Editorial Policy Checklist.

Statistics

For all statistical analyses, confirm that the following items are present in the figure legend, table legend, main text, or Methods section.

n/a	Confirmed	
☐	☒	The exact sample size (n) for each experimental group/condition, given as a discrete number and unit of measurement
☐	☒	A statement on whether measurements were taken from distinct samples or whether the same sample was measured repeatedly
☐	☒	The statistical test(s) used AND whether they are one- or two-sided<br>*Only common tests should be described solely by name; describe more complex techniques in the Methods section.*
☒	☐	A description of all covariates tested
☐	☒	A description of any assumptions or corrections, such as tests of normality and adjustment for multiple comparisons
☐	☒	A full description of the statistical parameters including central tendency (e.g. means) or other basic estimates (e.g. regression coefficient) AND variation (e.g. standard deviation) or associated estimates of uncertainty (e.g. confidence intervals)
☐	☒	For null hypothesis testing, the test statistic (e.g. F, t, r) with confidence intervals, effect sizes, degrees of freedom and P value noted<br>*Give P values as exact values whenever suitable.*
☒	☐	For Bayesian analysis, information on the choice of priors and Markov chain Monte Carlo settings
☒	☐	For hierarchical and complex designs, identification of the appropriate level for tests and full reporting of outcomes
☒	☐	Estimates of effect sizes (e.g. Cohen's d, Pearson's r), indicating how they were calculated

Our web collection on statistics for biologists contains articles on many of the points above.

Software and code

Policy information about availability of computer code

Data collection	No software was used
Data analysis	BWA, BWA-MEM, Picard, GATK, STAR, HMMcopy, GISTIC 2.0, Sequenza, BRASS, MAPSPLICE, TelSeq, ClusteredMutations, CTLPScanner, ESTIMATE, TIMER, DAVID v.8, MuSE, MuTect, Pindel, CLiP, custom codes described in Supplemental Methods.

For manuscripts utilizing custom algorithms or software that are central to the research but not yet described in published literature, software must be made available to editors and reviewers. We strongly encourage code deposition in a community repository (e.g. GitHub). See the Nature Portfolio guidelines for submitting code & software for further information.

Data

Policy information about availability of data

All manuscripts must include a data availability statement. This statement should provide the following information, where applicable:

- Accession codes, unique identifiers, or web links for publicly available datasets
- A description of any restrictions on data availability
- For clinical datasets or third party data, please ensure that the statement adheres to our policy

The exome and RNA sequencing data were deposited at the European Genome-phenome Archive under the study accession number: EGAS00001004575: https://ega-archive.org/studies/EGAS00001004575.

Field-specific reporting

Please select the one below that is the best fit for your research. If you are not sure, read the appropriate sections before making your selection.

☒ Life sciences ☐ Behavioural & social sciences ☐ Ecological, evolutionary & environmental sciences

For a reference copy of the document with all sections, see nature.com/documents/nr-reporting-summary-flat.pdf

Life sciences study design

All studies must disclose on these points even when the disclosure is negative.

Sample size	We did not use any statistical methods to determine sample size. Since this is a rare tumor, we collected all possible samples and conducted analyses on all.
Data exclusions	No data were excluded.
Replication	In vitro experiments were conducted in triplicate.
Randomization	N/A
Blinding	N/A

Reporting for specific materials, systems and methods

We require information from authors about some types of materials, experimental systems and methods used in many studies. Here, indicate whether each material, system or method listed is relevant to your study. If you are not sure if a list item applies to your research, read the appropriate section before selecting a response.

Materials & experimental systems

n/a	Involved in the study	
☐	☒	Antibodies
☐	☒	Eukaryotic cell lines
☒	☐	Palaeontology and archaeology
☒	☐	Animals and other organisms
☐	☒	Human research participants
☐	☒	Clinical data
☒	☐	Dual use research of concern

Methods

n/a	Involved in the study	
☒	☐	ChIP-seq
☒	☐	Flow cytometry
☒	☐	MRI-based neuroimaging

Antibodies

Antibodies used	ARID1A/BAF250A (Cell Signaling Technologies, D2A8U) and β-actin (Cell Signaling Technologies).
Validation	Validated by Cell Signaling Technologies using T-47D and Jurkat cell extracts where T-47D is negative for ARID1A.<br>Both antibodies were used in the manuscript: Loss of ARID1A Promotes Epithelial–Mesenchymal Transition and Sensitizes Pancreatic Tumors to Proteotoxic Stress<br>Hideo Tomihara, Federica Carbone, Luigi Perelli, Justin K. Huang, Melinda Soeung, Johnathon L. Rose, Frederick S. Robinson, Yonathan Lissanu Deribe, Ningping Feng, Mitsunobu Takeda, Akira Inoue, Edoardo Del Poggetto, Angela K. Deem, Anirban Maitra, Pavlos Msaouel, Nizar M. Tannir, Giulio F. Draetta, Andrea Viale, Timothy P. Heffernan, Christopher A. Bristow, Alessandro Carugo and Giannicola Genovese<br>Cancer Res January 15 2021 (81) (2) 332-343; DOI: 10.1158/0008-5472.CAN-19-3922

Eukaryotic cell lines

Policy information about cell lines

Cell line source(s)	JN-DSRCT provided from Dr. Kikuchi's laboratory (Fukuoka University, Fukuoka, Japan)
Authentication	Identified twice per year in MDA characterized cell line core (CCLC) using short tandem repeat (STR) fingerprinting with an AmpFLSTR Identifier kit.
Mycoplasma contamination	Tested negative for mycoplasma contamination using the MycoAlert Detection Kit (Lonza Group Ltd.) according to the manufacturer's protocol.

Commonly misidentified lines<br>(See ICLAC register)	None.

Human research participants

Policy information about studies involving human research participants

Population characteristics	15 patients who were diagnosed with DSRCT. The median age of diagnosis is 18.7. All patients were male, no female patients were included. 13 samples were from the primary tumors. 2 were metastatic. 14 patients had known prior treatment with chemotherapy, surgery, and/or radiation.
Recruitment	Patients diagnosed with DSRCT were asked if they would like to participate in research. provided their written informed consent for their samples to be deidentified and to be used in research
Ethics oversight	MD Anderson Cancer Center Institutional Review Board

Note that full information on the approval of the study protocol must also be provided in the manuscript.

Clinical data

Policy information about clinical studies

All manuscripts should comply with the ICMJE guidelines for publication of clinical research and a completed CONSORT checklist must be included with all submissions.

Clinical trial registration	N/A
Study protocol	N/A
Data collection	N/A
Outcomes	N/A
